# Neutral Formazan Ligands Bound to the *fac*-(CO)_3_Re(I) Fragment: Structural, Spectroscopic, and Computational Studies

**DOI:** 10.1021/acs.inorgchem.2c02168

**Published:** 2022-08-15

**Authors:** Liliana Capulín Flores, Lucas A. Paul, Inke Siewert, Remco Havenith, Noé Zúñiga-Villarreal, Edwin Otten

**Affiliations:** † Stratingh Institute for Chemistry, University of Groningen, Nijenborgh 4, 9747 AG Groningen, The Netherlands; ‡ Instituto de Química, Universidad Nacional Autónoma de México, Ciudad Universitaria, Circuito Exterior, 04510 México, D.F., México; § Universität Göttingen, Institut für Anorganische Chemie, Tammannstraße 4, D-37077 Göttingen, Germany

## Abstract

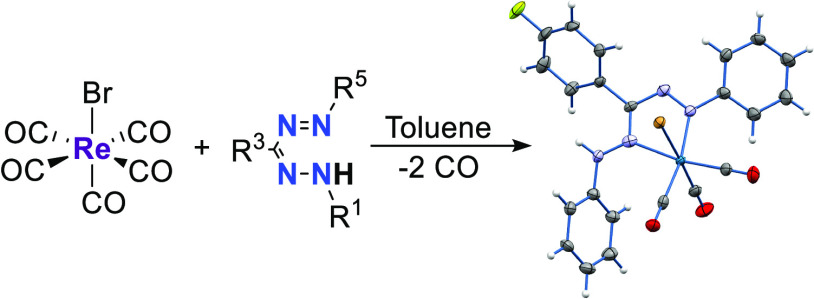

Metal complexes with ligands that coordinate via the nitrogen atom of azo (N=N) or imino (C=N) groups are of interest due to their π-acceptor properties and redox-active nature, which leads to interesting (opto)electronic properties and reactivity. Here, we describe the synthesis and characterization of rhenium(I) tricarbonyl complexes with neutral *N*,*N*-bidentate formazans, which possess both N=N and C=N fragments within the ligand backbone (Ar^1^-NH-N=C(R^3^)-N=N-Ar^5^). The compounds were synthesized by reacting equimolar amounts of [ReBr(CO)_5_] and the corresponding neutral formazan. X-ray crystallographic and spectroscopic (IR, NMR) characterization confirmed the generation of formazan-type species with the structure *fac*-[ReBr(CO)_3_(κ^2^-*N*^2^*,N*^4^(Ar^1^-N^1^H-N^2^=C(*R*^3^)-N^3^=N^4^-Ar^5^))]. The formazan ligand coordinates the metal center in the ‘*open*’ form, generating a five-membered chelate ring with a pendant NH arm. The electronic absorption and emission properties of these complexes are governed by the presence of low-lying π*-orbitals on the ligand as shown by DFT calculations. The high orbital mixing between the metal and ligand results in photophysical properties that contrast to those observed in *fac*-[ReBr(CO)_3_(L,L)] species with α-diimine ligands.

## Abstract 2

Tricarbonyl rhenium complexes with formazan ligands are synthesized, and their (electronic) structures were elucidated using crystallographic, spectroscopic, and computational methods.

## Introduction

**Scheme 1:**
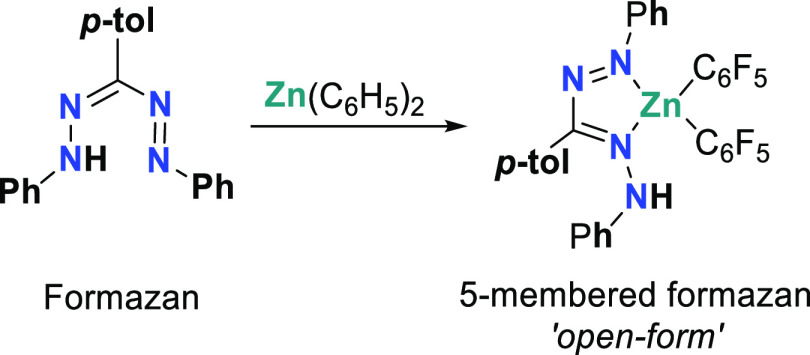
Synthesis of Zn Formazan Species

Formazans are a large family of compounds containing the R^1^-NH-N=C(R^3^)-N=N-R^5^ backbone, known for their use as analytical reagents for metal detection^1^ and as cellular^2^ and textile dyes.^3^ These applications are the consequence of its well-defined redox chemistry^4^ and its ability to chelate metal centers in its deprotonated form, i.e., the delocalized formazanate anion (R^1^-N=N-C(R^3^)=N-N-R^5^)^−^. Although formazanate coordination chemistry was first described in 1941,^5^ it was not until the last decade that its study has reemerged due to its electrochemical and optical properties.^6^ A wide variety of formazanate complexes with both main group and transition metal elements have been reported, wherein the (anionic) ligand usually coordinates through the terminal donor sites to form 6-membered chelates.^7^ In addition to taking advantage of the unique optoelectronic properties imparted by formazanate ligands, recent reports show that their redox-active nature can also be used to obtain new catalytic reactivity.^8^ In contrast to complexes with anionic formazanates, reports on coordination of the neutral formazan fragment remain scarce to date. In 2015, our group described the first example of a formazan-type complex,^9^ in which the neutral ligand binds Zn(C_6_F_5_)_2_ through one terminal and one internal nitrogen atoms yielding a five-membered chelate (Scheme 1), also described as the *‘open’* coordination mode. It was hypothesized that the poor basicity of the Zn-C_6_F_5_ group in the precursor allowed the isolation of the Zn-formazan compound, as the more basic reagent ZnMe_2_ does result in rapid deprotonation of the formazan NH group.^9^

Metal complexes with ligands containing the NH functionality have gained importance in catalysis since the NH arm can serve as an anchor for substrate recognition, thus enhancing catalyst selective and activity.^10^ A proton source located at the proximity of the metal center has been widely investigated in the proton-coupled electron transfer reduction of small molecules relevant in energy conversion reactions such as hydrogen evolution^11,12^ and CO_2_ reduction.^13−15^ It was proposed to modulate the redox properties, aid in the stabilization of intermediates, or impact the kinetics due to the increased local proton concentration. Furthermore, deprotonation of the NH group is known to modify the electronic and geometric structure of such complexes.^16−18^

**Chart 1:**
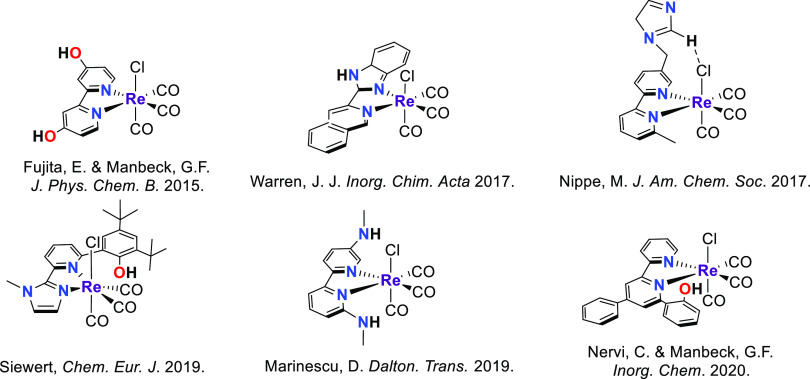
Representative Examples of Re Complexes Bearing an XH Functionality

Extensive research has focused on the properties and potential applications of *fac*-[L,LReX(CO)_3_] (L = α-diimine) compounds in medicinal inorganic chemistry,^19,20^ material science,^21,22^ and catalysis.^23,24^ Particularly, these compounds have shown to be good candidates for electrochemical CO_2_ reduction, in which a proton source is required either for enhancing or triggering the catalytic process.^25,26^ Mainly, Re-based bipyridine systems have been reported as catalysts for CO_2_ electroreduction, wherein the presence of XH (X = O, N, C)^18,27,28^ functionalizations boosts the catalytic effect or induces other reactivity patterns (Chart 1).^29−31^ Key to the catalytic conversion of CO_2_ to CO by the well-studied bipyridine Re and Mn complexes is the involvement of the supporting (‘redox-active’) ligand in the reduction chemistry.^28,32−34^ We hypothesize that replacing the bipyridine ligand (an aromatic α-diimine) for a redox-active formazan ligand (formally an amino-substituted α-azoimine) could provide an avenue to influence the potential at which reduction of the catalyst occurs.^35,36^ In addition, such ligands provide access to flexible coordination modes (hemilability)^37^ due to the presence of four nitrogen atoms in the backbone, as well as proton-responsivity via the NH moiety that is in close proximity to the metal center, features that are key to the activity/selectivity of metalloenzymes but challenging to emulate in synthetic catalysts.^38^

Herein, we report synthesis of a series of *fac*-Re(CO)_3_(formazan) complexes and investigate their (electronic) structures and photophysical properties.

## Results and Discussion

### Ligand Synthesis

**Scheme 2:**
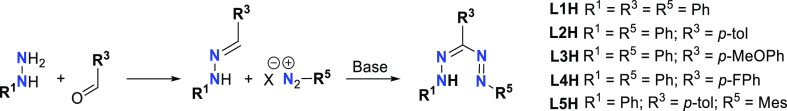
Synthesis of Formazan Ligands

Formazan ligands **L1H**–**L4H** were synthesized according to the procedure reported by Hicks and coworkers, via aldehyde condensation with phenylhydrazine followed by a coupling reaction with phenyldiazonium chloride in a biphasic reaction medium (CH_2_Cl_2_/water) under mild basic conditions.^4^ Similarly, **L5H** was prepared using the methodology previously described by our group, in which the coupling step is carried out in acetone/water with NaOH as base (Scheme 2).^9^ In all cases, the compounds were obtained in moderate yields after purification (27–54%).

### Complex Synthesis

**Scheme 3:**
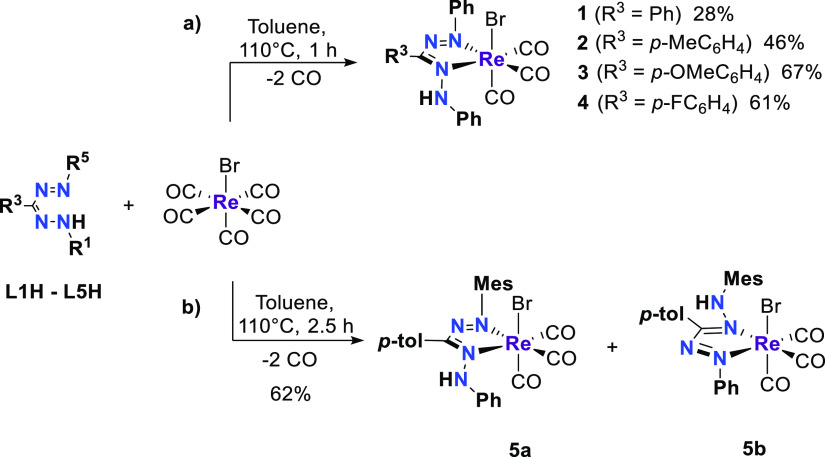
General Synthesis of Compounds (a) **1**–**4** and (b) the Mixture of Isomers **5a** and **5b**

Equimolar amounts of [ReBr(CO)_5_] and the corresponding formazan, **L1H–L4H**, reacted in refluxing toluene for 1 h to afford complexes **1**–**4** in moderate to good yields (28–67%) (Scheme 3a). In all cases, complete conversion of the starting material was confirmed by ^1^H NMR and infrared spectroscopy. The compounds are air-stable solids with dark red color and are soluble in low to medium polarity solvents. Complexes **2–4** were isolated as pure materials by either recrystallization or rinsing with pentane. A minor impurity was invariably present (^1^H NMR spectroscopy) in the isolated material of **1**. Attempts to further purify the material by crystallization were unsuccessful. The reaction of the asymmetric formazan **L5H** with [ReBr(CO)_5_] in refluxing toluene gave a mixture of two complexes (**5a** and **5b**) based on ^1^H NMR spectroscopy (Scheme 3b), which differ in the substituent at the NH position (Mes or Ph). Unsurprisingly, complexes **5a/b** present similar physical properties—dark red solids soluble in low polarity solvents—that we were unable to separate, and solution characterization data are reported below for the mixture.

### FT-IR Spectroscopy

**Figure 1:**
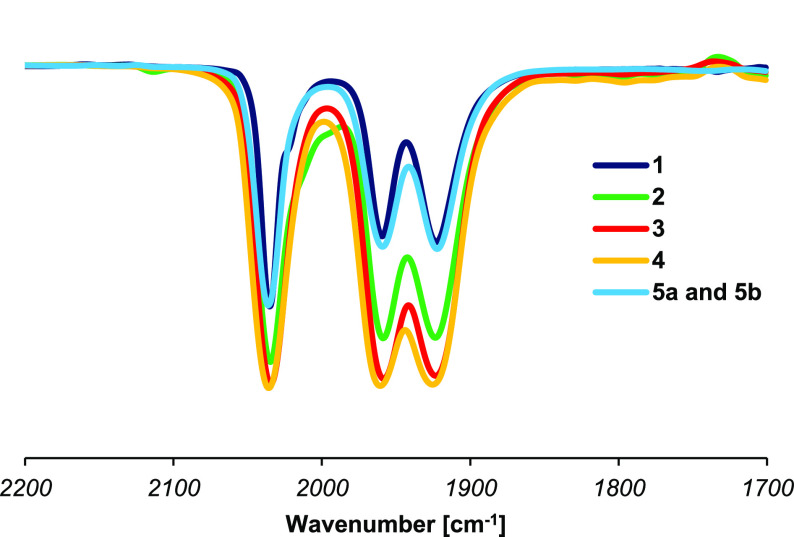
CO stretching bands (ν(CO)) in the FT-IR spectra of complexes **1**–**4** and the mixture **5a** and **5b** in CH_2_Cl_2_ solution at rt.

The infrared spectra of complexes **1**–**4** and the mixture **5a**/**b** feature the characteristic pattern for *fac*-tricarbonyl species: three intense bands in the ν(CO) carbonyl region arising from the IR-active 3A normal vibration modes for complexes with a *C*_1_ symmetry (Figure 1). The CO stretching frequencies for compound **1** are observed at 2035, 1959, and 1923 cm^–1^ in CH_2_Cl_2_ solution. The effect of the *para*-substituent on the aromatic ring (R^3^) is minimal, and compounds **2**–**5** show virtually identical IR spectra.

### NMR Studies

^1^H NMR spectra of **1–4** show a singlet ca. 8.5 ppm assigned to the hydrazo proton, consistent with the presence of a neutral formazan ligand (see Figure S1a–S4a). In agreement with the presence of an asymmetric, protonated formazan ligand, the ^13^C NMR spectrum shows three distinct carbonyl resonances between 185 and 192 ppm indicative of *C*_1_ symmetry for these Re complexes. In ^13^C NMR spectroscopy, the N-Ph *ipso*-carbon atoms attached to the formazan backbone display distinctive chemical shifts. Unequivocal assignment of these was possible based on an HSQC experiment where coupling between the hydrazo NH group and one of the Ph *ipso*-C resonances was observed. The resonance of the *ipso*-C bound to the azo group appears at 157 ppm, which is downfield from both the *ipso*-C linked to the NH function located ca. 141 ppm, and the *ipso*-C of the C-Ar group (120–125 ppm). Similarly, the N-Ph groups are inequivalent in the ^1^H NMR spectrum also at elevated temperature (80 °C in toluene-*d*_8_), suggesting that chemical exchange by proton transfer between the azo (C-N=N-Ph) and hydrazo (C=N-NH-Ph) fragments does not readily occur. This was further corroborated by the absence of exchange crosspeaks in the 2D EXSY spectrum (80 °C, mixing time of 0.5 s, Figure S7).

**Figure 2:**
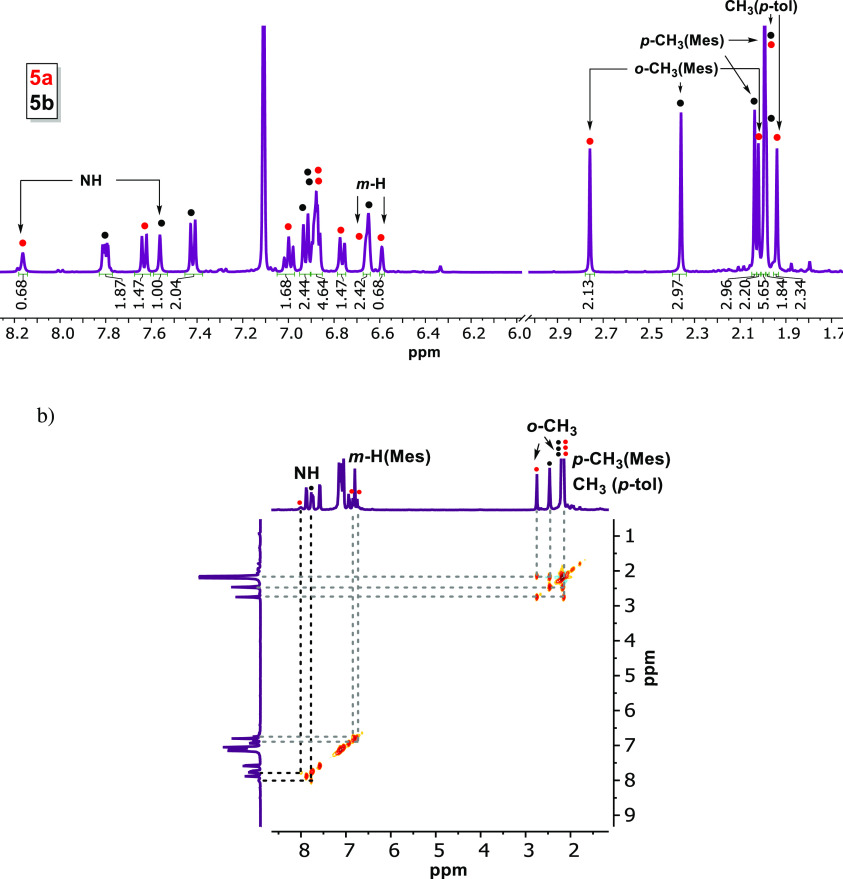
(a) ^1^H NMR spectrum of the mixture **5a** and **5b** at room temperature in benzene-*d*_6_. (b) EXSY experiment at 80 °C in toluene-*d*_8_.

^1^H NMR analysis of the reaction mixture of complexes **5a/b** indicates the generation of two main products in ratio of 0.7:1.0 with both compounds exhibiting the characteristic NH proton signal of the neutral formazan ligand at 8.15 and 7.60 ppm, respectively (Figure 2a). Minor signals for another species were observed (<5%) but not investigated further. For the two major products, the observation of eight unique resonances in the aliphatic region of the ^1^H and ^13^C NMR spectra indicates that all CH_3_ groups are inequivalent: each of the two products features four signals due to the CH_3_ substituents at the *p*-tolyl (1) and mesityl (3) rings. Thus, at room temperature, the rotation around the N-Mes bond is slow on the NMR timescale. In the most downfield part of the ^13^C{^1^H} NMR spectrum, i.e., between 180 and 200 ppm, there are six resonances that can be attributed to carbonyl ligands, which corroborates that both **5a** and **5b** are tricarbonyl rhenium complexes (Figure S6). Based on the spectroscopic data, we assign **5a** and **5b** as two different isomers with the composition [(**L5H**)Re(CO)_3_Br], which differ in the nature of the ‘pendant’ (non-coordinating) N-Ar group of the formazan (see Scheme 3b). Heating an NMR tube containing the mixture of complexes **5a/b** to 80 °C inside the NMR spectrometer did not significantly change their molar ratio. Inspection of 2D NMR experiments allowed the assignment of ^1^H and ^13^C spectra (see Figure S6). Identification of the *m*-CH (Mes) and the *m*- and *o*-CH (*p*-tolyl) protons allowed establishing of the connectivity in both of the isomeric compounds present in solution. The ^1^H,^13^C correlations in the HMBC spectrum between the NH fragment and the carbon atoms that are two and three bonds away indicate that in the major isomer (**5b**), the NH group is bound to a mesityl group, whereas in **5a** it is connected to a phenyl group. The greater shielding effect of mesityl compared to the phenyl group causes the NH proton of the former to appear at higher field (δ 7.61 ppm in **5b** and 8.22 ppm in **5a**).

To investigate the dynamics of isomers **5a**/**b** in solution, we collected a ^1^H EXSY NMR spectrum at 80 °C in toluene-*d*_8_ (Figure 2b). Crosspeaks are observed between the Mes *ortho-*CH_3_ groups within each isomer due to rotation around the N-Mes bond but not between isomers **5a** and **5b**. Whereas free formazans undergo intramolecular proton exchange rapidly (‘tautomerization’),^39,40^ the lack of exchange between **5a**/**b** indicates that the Re–N bonds are non-labile and coordination to the Re center effectively blocks exchange. This is in agreement with the data for the symmetrical derivative **4**, which also does not show exchange between the azo and hydrazo fragments (*vide supra*). It should be noted however that the EXSY spectrum does evidence exchange between the NH groups in **5a**/**b**, which we believe to occur by an intermolecular pathway instead. This is further corroborated by the observation of exchange crosspeaks between the NH protons in **5a**/**b** and residual H_2_O when the NMR solvent is not fully anhydrous (Figure S8).

Additional experiments were conducted to determine whether intermolecular proton interchange processes also place in the complexes containing a symmetric formazan ligand. Partial H/D exchange of the NH proton in complex **4** was achieved by mixing a CDCl_3_ solution of the aforementioned complex with D_2_O. ^1^H NMR analysis of the resulting mixture indicated that it was composed of 60% **4D** (deuterated product) and 40% the non-deuterated species **4**. The remaining D_2_O was subsequently removed by stirring over MgSO_4_, the **4**/**4D** mixture was isolated and then reacted with an equivalent of **2** in C_6_D_6_. Monitoring the composition by ^1^H NMR spectroscopy showed that the intensity of the NH resonance of **4** increased (to 72%) in the course of 30 min with a concomitant decrease of that in **2**, confirming that intermolecular proton exchange is taking place (see Figure S9).

### Structural Studies

**Figure 3:**
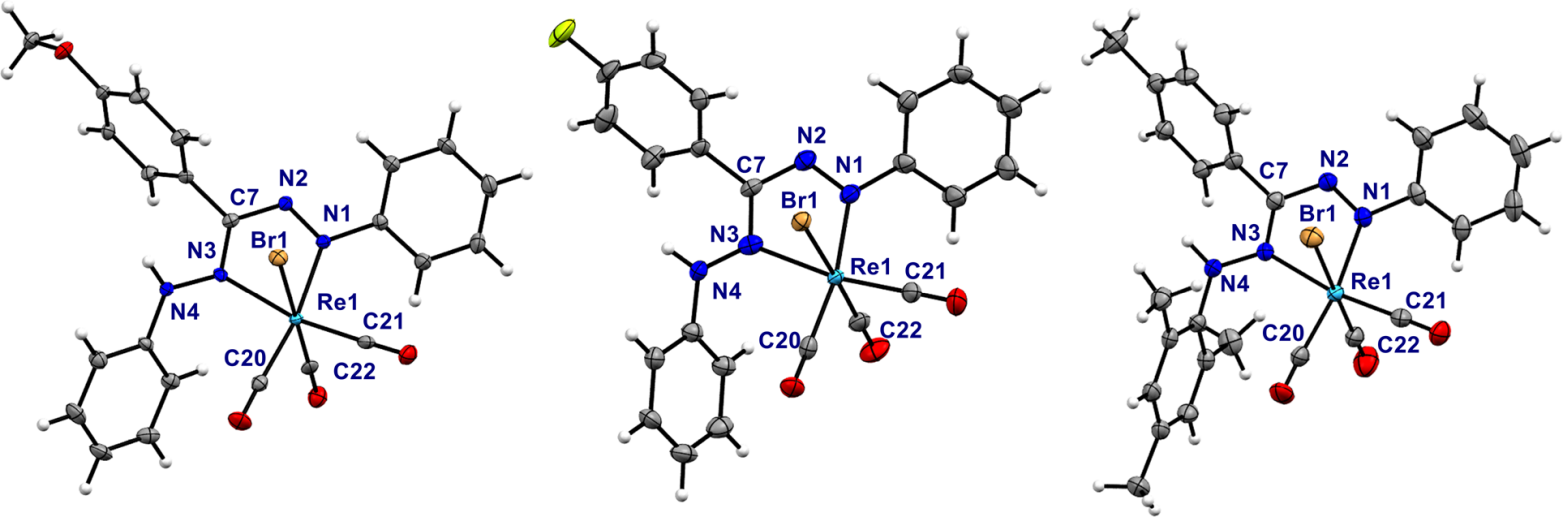
Molecular structures of **3** (left), **4** (middle), and **5b** (right) showing 50% of ellipsoids.

Crystals suitable for single-crystal X-ray diffraction were obtained from slow diffusion of pentane into a CHCl_3_ solution of compounds **3** and **4**, respectively. The mixture of **5a**/**b** did not crystallize using the same method, but we were able to obtain a microcrystalline sample from hot hexane that contained some small needles that were suitable for X-ray crystallographic characterization. This was identified as isomer **5b**, in which the sterically most demanding Mes group is situated at the non-coordinated N atom of the ligand; the solid-state structure observed for this material is consistent with the major species in solution by NMR spectroscopy. Analysis of the molecular structures of **3**, **4**, and **5b** shows that the three compounds are isostructural (see Figure 3 and Table 1 for pertinent bond lengths and angles). The geometry around the metal center is pseudo-octahedral with the carbonyl ligands in a facial arrangement. The formazan fragment coordinates in a bidentate fashion through atoms N1 and N3, generating a five-membered chelate. Coordination of the neutral formazan is scarce, only observed in the complex [**L2H**]Zn(C_6_F_5_)_2_ previously reported by our group.^9^ The formazan bite angles are virtually identical in the three complexes (**3** = 73.04(9)°, **4** = 73.0(2)°, **5b** = 72.9(1)°) and somewhat smaller than the bite angle reported for the [**L2H**]Zn(C_6_F_5_)_2_ complex (74.23(13)°). The C7–N2 and C7–N3 bond lengths are different from each other, the magnitude of the C7−N3 bond lies in between the typical values for C–N single and double bonds (−C(sp^2^)–N– = 1.355 Å; −C(sp^2^)=N– = 1.279 Å), while the C7−N2 bond length indicates a single bond character. The N1–N2 bond length is longer than a N=N double bond (−N=N– = 1.240 Å) and smaller than a N–N single bond (−N–N– = 1.425 Å).^41^ The metallacycle is not fully planar as the Re atom is displaced out of the ligand plane (N1–N2–C7–N3) by 0.213–0.393 Å. The dihedral angle between the ligand plane and a phenyl group in the R^1^ position is similar in complexes **3** (50.67°) and **4** (51.12°). Changing the R^1^ substituent for the bulkier mesityl group (**5b**) causes a rotation out of the ligand plane by almost 30° resulting in a dihedral angle of 79.52° that prevents steric interactions between the Mes substituent and the equatorial CO ligand. The structure indicates that rotation around the N-Mes bond cannot occur freely due to these steric interactions, which is in agreement with the solution NMR data discussed above. The Re1–N1 bond length to the azo moiety is virtually the same in the three complexes (2.099–2.126 Å) but it is shorter than the Re–N(azo) bond length reported for the related [ReBr(CO)_3_(azopyridine)]^42^ complex (2.156(3) Å). The Re1–N3 bond lengths are in accordance with the typical Re–N(imine) bond distances (2.173–2.185 Å in compounds **3**–**5***vs* 2.173(3) Å in [ReBr(CO)_3_(6-methoxipyridine-2-yl)-*N*-(2-methylthiophenyl)methanimine)], respectively).^43^ The unusually short Re–N1(azo) bond length reflects that π-backdonation from the Re center to the azo group is more pronounced in the formazan species than in azopyridine complexes.^44^ The π-acceptor capabilities of the azo ligand are also reflected in the Re–carbonyl bond lengths. The Re–C20 bond length, *trans* to the azo group, is longer than the Re–C21 bond length. This is consistent with the considerable π-acidity of the azo group,^45,46^ which appears to be more significant in our formazan complexes than in the corresponding azopyridine analogues.^42^

**Table 1:** Selected Metrical Parameters for **3**, **4**, and **5b** (Bond Lengths in Å, Angles in °)

	**3**	**4**	**5b**
Re1–Br1	2.5977(3)	2.6236(7)	2.5946(6)
Re1–N1	2.126(2)	2.099(6)	2.122(3)
Re1–N3	2.173(3)	2.185(5)	2.174(4)
Re1–C20	1.955(3)	1.955(6)	1.957(5)
Re1–C21	1.921(3)	1.919(5)	1.916(6)
Re1–C22	1.941(2)	1.918(6)	1.964(4)
N1–N2	1.298(3)	1.291(7)	1.293(5)
C7–N2	1.363(4)	1.382(7)	1.364(8)
C7–N3	1.330(4)	1.319(8)	1.326(5)
N3–N4	1.343(4)	1.324(8)	1.343(7)
Br1–Re1–C22	178.02(9)	176.8(2)	178.6(2)
N1–Re1–C20	170.3(1)	168.7(2)	170.2(2)
N3–Re1–C21	166.5(1)	169.7(2)	170.3(2)
N1–Re1–N3	73.04(9)	73.0(2)	72.9(1)

### UV–vis Spectroscopy

**Figure 4:**
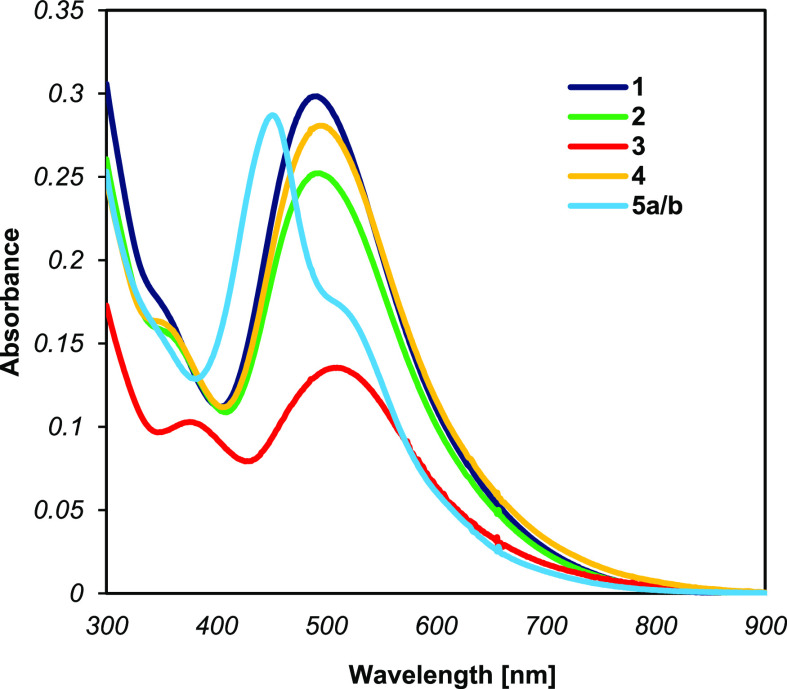
Absorption spectra for compounds **1**–**4** and **5a**/**b** in toluene solution.

The electronic spectra of complexes **1–4** and the mixture of complexes **5a**/**5b** were measured at 25 °C in toluene (*c* ≈ 10^–5^ M), Figure 4. Their corresponding data are summarized in Table 2. Compounds **1–4** show similar features: an intense band in the range of 490–510 nm with maximum molar absorptivities from 18,000 to 28,000 M^–1^ cm^–1^. DFT calculations and comparison to literature data allows us to assign this band to electronic excitations that are Re(d_π_) → azo(π*)^42^ MLCT and formazan π → π* in nature.^9^ A band of lower intensity in the range of 330–400 nm (ε =15,000–16,500 M^–1^ cm^–1^) is observed in all compounds. While bands at similar energies are typically assigned to metal-to-ligand charge transfer in related compounds,^47,48^ time-dependent DFT calculations for **4** instead indicate little involvement of the Re d-orbitals in this transition (*vide infra*). Overall, the λ_max_ values of **1**, **2**, and **4** do not differ significantly, suggesting that the substituent at the *para*-position of the N-Ar rings has little influence on the energy of the electronic transitions, which is in line with the notion that the acceptor orbital in these transitions is a formazan π*-orbital that is relatively insensitive to the *para*-substituent. In the case of complex **3**, λ_max_ is slightly red-shifted (509 nm) compared to complex **1** (490 nm), indicating that the involvement of the π-donating OMe group on the conjugated system is noticeable_._ When the spectrum of **4** was recorded in acetonitrile, a modest hypsochromic shift was observed (λ_max_^AcCN^ = 483 nm (Figure 6a); λ_max_^Toluene^ = 495 nm), showing that these species manifest a small, negative solvatochromism. Comparing λ_max_ values to those reported for complexes with anionic formazanate ligands,^9,49,50^ the absorption maxima in **1**–**4** are blue-shifted due to a smaller extent of π-conjugation within the backbone of the neutral ligands compared to the fully delocalized anions. The mixture of complexes **5a**/**b** features two intense bands at 520 and 452 nm derived from the MLCT and π–π* formazan electronic transitions and a shoulder at 350 nm. Overall, the influence of the substituents on the lowest energy band is more pronounced when they are located at the N=N and NH formazan positions, similar to what was observed in complexes with anionic formazanate ligands.^6^ Clearly, the nature of electronic absorptions for the two isomers **5a**/**b** is quite distinctive, as is manifested by the significant shift in λ_max_.

**Table 2:** UV–vis Absorption Data

compound	λ_max_ (nm)	ε (M^–1^·cm^–1^)	λ_max_ (nm)	ε (M^–1^·cm^–1^)
**1**	356	16,500	490	18,100
**2**	353	15,300	485	19,400
**3**	375	10,500	509	14,100
**4**	353	15,300	495	27,000
**5a**/**b**			452	
			∼520^a^	

a Approximate band position due to its appearance as a shoulder on the more intense absorption of the other isomer.

### Density Functional Theory (DFT) Calculations

For representative complexes **4** and **5a**/**b**, geometry optimizations were carried out in the ground state using density functional theory (DFT; MN15L^51^ functional and def2-TZVP^52^ basis set) using the crystallographic coordinates as a starting point. The geometries were confirmed to be minima on the potential energy surface by frequency calculations (no imaginary frequencies); the resulting structures are in good agreement with the metrical parameters obtained from X-ray diffraction (complexes **4** and **5b)**, albeit that the Re–Br and hydrazo N–N bonds are slightly overestimated (Tables S2 and S3). Analysis of the frontier orbitals at the optimized geometry of **4** showed that the HOMO is mainly localized on the [ReBr(CO)_3_] core and is composed of a Re *d*_π_ orbital that is antibonding with a bromine *p* orbital and π-bonding with the CO ligand located *trans* to Br. The HOMO level also contains some ligand character (the hydrazo-phenyl fragment). On the other hand, the LUMO is primarily a π*-orbital of the formazan framework, with a minor Re 5*d* character (Figure S10).

The optimized structures of **5a** and **5b** are overall similar, but the variation in the position of the Mes group (on the *azo* or *hydrazo* N-atom, respectively) leads to somewhat different frontier orbitals. While those of **5b** are similar to **4**, the HOMO of **5a** has noticeably smaller formazan contribution (Figures S12 and S14). The relative stability between the isomeric forms **5a** and **5b** was also evaluated based on these DFT calculations. Using the gas phase geometries, the Gibbs free energy difference between both compounds was computed in toluene solution using the solvation energies from SMD calculations and found to be 1.6 kcal/mol at room temperature, with **5b** being the most stable isomer. Qualitatively, the trend in relative stability is consistent with our empirical data since compound **5b** is the predominant species in the reaction mixture according to the NMR integration. It should be noted that it is also possible that the **5a**/**5b** ratio found experimentally is kinetically controlled as no interconversion between both isomers was observed.

Time-dependent density functional theory (TDDFT) calculations were carried out on complex **4** as a representative example. Relevant excitations were analyzed in more detail using natural transition orbital calculations (NTOs) to provide insight into their nature. According to the calculations, the three lowest-energy transitions in **4** (λ_calc_ = 645, 539 and 526 nm) all have small oscillator strength and involve transitions from orbitals centered on the [ReBr(CO)_3_] fragment (π*(Re-Br) and (π(Re-CO)) into the formazan π*-orbital. The fourth excited state, with the highest oscillator strength in the visible range (λ_calc_ = 487 nm; λ_max,exp_ = 495 nm, Figure S11a-b), has a more pronounced formazan (intraligand) π–π* character, involving an occupied azo π-orbital as the donor (see Table S4 for the corresponding NTO), but also here the contribution of metal-based orbitals is still clearly noticeable. Thus, in all excitations in the visible range, there is extensive mixing between the metal and ligand orbitals in the ground and excited states, which results in electronic transitions of mixed nature: all show contributions from MLCT Re(dπ) → formazan(π*), LLCT Br(*p*) → formazan(π*), and ILCT azo(π) → formazan(π*) excitations. The strong metal–ligand orbital mixing results in reduced charge transfer character in the MLCT bands, which is reflected in the minor influence of solvent polarity (toluene *vs* acetonitrile) on the empirical electronic absorption spectrum (*vide supra*).^53^ Similar to the absorptions in the visible range, analysis of the NTOs of the higher energy transitions shows that these involve the formazan π*-orbital as the acceptor and are also highly mixed in character.

**Figure 5:**
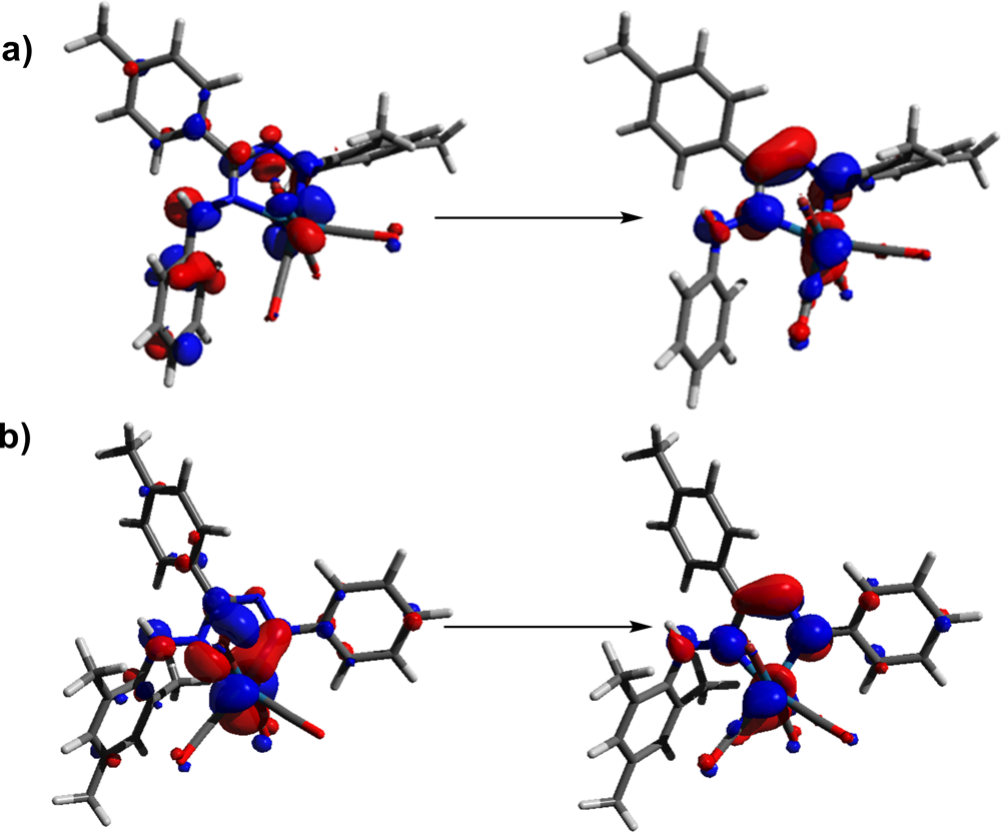
Natural transition orbitals for the highest-intensity excitation in the visible (isovalue = 0.05) for (a) **5a** and (b) **5b** represented as a hole → electron.

TDDFT calculations were performed on the optimized structures of complexes **5a/b** to understand the impact of the pendant R^1^/R^5^ arms on the electronic transitions. The intense low-energy absorption is computed to be shifted to higher energy for **5a** (444 nm) compared to **5b** (489 nm), see Figures S13 and S14, respectively. This is in agreement with the empirical UV/vis spectrum, which shows two distinct bands at 452 and 520 nm for the **5a**/**b** mixture. As in **4**, the natural transition orbital pair for the main low-energy excitation in **5b** consists of a ‘hole’ NTO on the [ReBr(CO)_3_] core, whereas the excited electron (‘particle’ NTO) consists primarily of the π* formazan orbital. A comparison of the NTOs for **5a** and **5b** shows that the main difference between the two isomers is found in the hole NTO (Figure 5), which has a higher formazan contribution in **5a**. Based on the optimized geometries, the orientation of the azo-NAr ring changes upon swapping the aryl groups on the nitrogen atoms (Ph/Mes): the angle between the plane defined by the five-membered chelate ring and the Mes-substituent is 73.77° in **5a**, whereas the corresponding angle with the Ph-substituent in **5b** is only 39.28°. To test our hypothesis that the orientation of the azo-NAr group has a major impact on the spectral properties, we took the geometry of **5b** and rotated the N-Ph group out of the ligand plane to be in the same orientation as the N-Mes group in **5a**. This structure is labeled **5b_rot**. The main visible band in the TDDFT spectrum calculated at the **5b_rot** geometry is blue-shifted by 30 nm (1342 cm^–1^) compared to **5b**, but the other transitions remain at similar energies (Figure S17). An analysis of the orbital mixing between the azo-NAr ring and the rest of the ligand π-system confirms that rotating the Ar ring out of the plane disrupts conjugation (Table S7), and thus we conclude that this is responsible for the spectral shift observed.

### Luminescence Spectroscopy

**Figure 6:**
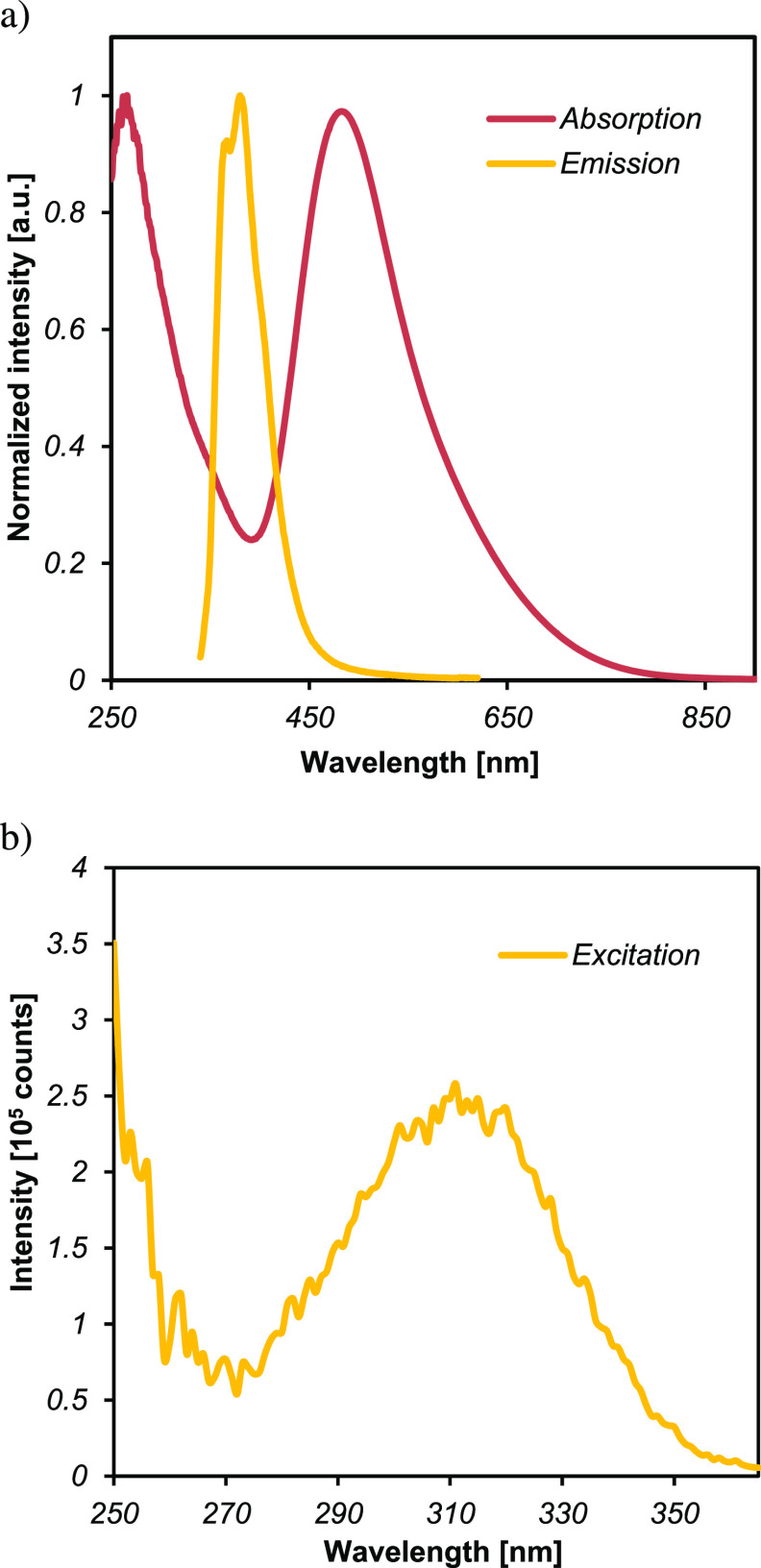
(a) Normalized absorption and emission spectra of **4** in MeCN recorded at λ_exc_ = 320 nm; (b) excitation spectrum of **4** in MeCN. The data were collected at room temperature.

The emission spectrum of **4** measured at room temperature in acetonitrile using an excitation wavelength of 320 nm shows a broad featureless emission band at 380 nm (Figure 6a). The excited state showed a monoexponential decay (λ_ex_ = 370 nm) with a lifetime (τ) of 3.69 ns under a N_2_ atmosphere, which does not appreciably change under O_2_ (τ = 3.60 ns) (see Figure S19). Furthermore, the excitation spectrum (λ_em_ = 380 nm) exhibits a broad band centered at 310 nm (Figure 6b). In contrast to the majority of *fac*-[ReX(CO)_3_(L,L)] complexes with bidentate N-donor ligands (e.g., α-diimines), which typically show emission at higher wavelengths (400–600 nm),^54,55^ this data shows that the triplet (metal–ligand or ligand-centered)^56^ excited states typical for the photoluminescence of *fac*-[ReX(CO)_3_(L,L)] compounds^57^ are non-emissive in formazan Re(I) species. This also stands in contrast to complexes with anionic formazanate ligands, which show highly tunable emission with large Stokes shifts at much lower energies.^58−60^

## Conclusions

We described the straightforward synthesis of the first complexes bearing the neutral formazan ligand toward a group 7 metal. The *fac*-(CO)_3_ReBr(formazan) complexes obtained contain a five-membered chelate ring, in which the ligand binds via the nitrogen atoms of the azo (N=*N*-Ar) and hydrazo (C=*N*-NHAr) groups, which leaves a pendant acidic (exchangeable) NH moiety in close proximity to the metal center. Structural and spectroscopic data demonstrate that the formazan ligand is tightly bound to the metal center, which is due to the strong π-acceptor character of the ligand. The effect of ligand substituents on the properties of the complexes is only minor, but the preparation of an asymmetric derivative with a N-Mes substituent demonstrates that changes in the sterics shift the electronic absorption spectrum due to changes in the conjugation within the ligand. Computational studies at the DFT level confirm a high degree of covalency in the metal-formazan interaction and highly mixed metal–ligand character of the frontier orbitals, which is sensitive to the degree of conjugation within the ligand as demonstrated by sterically switching ‘off’ π-interactions in the derivative with a N-Mes group (compound **5a**). Unlike many *fac*-[ReX(CO)_3_(L,L)] compounds (L,L = α-diimine ligands) reported in the literature, our formazan complexes are only weakly luminescent in the near-UV (λ_em_ = 380 nm), and emission from the lower-energy excited states is not observed. In ongoing work, we are investigating the possibility of using the proton-responsive nature of the NH group (i.e., formazan/formazanate interconversion) in ‘cooperative’ reactivity of this type of complexes.

## Experimental Section

### General Considerations

All work—except ligand synthesis—was conducted under a nitrogen atmosphere using conventional Schlenk and vacuum-line techniques. Pentane and toluene (Aldrich, anhydrous. 99.8%) were passed over columns of Al_2_O_3_ (Fluka) and BASF R3-11-supported Cu oxygen scavengers. [ReBr(CO)_5_] was prepared according to the published procedures^61^ from [Re_2_(CO)_10_] (Aldrich, 98%) and Br_2_ (Aldrich, 98%). The ligands 1,5-diphenyl-3-*p*-tolylformazan (**L2H**), 1,5-diphenyl-3-*p*-methoxyphenylformazan (**L3H**), and 1,5-diphenyl-3-*p*-flourophenylformazan (**L4H**) were synthesized using the methodology reported by Hicks and coworkers.^4^ Particularly, 5-mesityl-1-phenyl-3-*p*-tolylformazan (**L5H**) was prepared using a modified procedure published by our group.^9^ 1,3,5-Triphenylformazan (**L1H**, TCI, 92%), *p*-tolualdehyde (Aldrich 97%), 4-methoxybenzaldehyde (Aldrich, 98%), 4-fluorobenzaldehyde (Aldrich, 98%), phenylhydrazine (Aldrich, 99%), aniline (Aldrich 99%), sodium nitrite (Aldrich, 99%), sodium carbonate (Aldrich, 99.5%), and [NBu_4_]Br were used as received. CHCl_3_ (Aldrich, 99%) and CDCl_3_ (Aldrich, 99.8 atom %D) were used without further purification.

NMR spectra were measured on Mercury 400, Varian Inova 500, or Bruker 600 MHz spectrometers. Residual solvent signals were used as internal reference for ^1^H and ^13^C spectra and reported in ppm relative to TMS (0 ppm). Complete assignments were based on two-dimensional experiments (COSY, HSQC, HMBC) using standard pulse sequences. FT-IR spectra were collected in DCM solution on a JASCO 4700 series FT-IR spectrometer in transmission mode using a liquid cell with CaF_2_ windows. UV–vis spectra were recorded in toluene solution on an Agilent Technologies 8453 UV–vis spectrophotometer. Luminescence spectroscopy were measured on a Fluorolog-3 spectrometer from HORIBA Jobin Yvon.

X-ray diffraction data were collected at 100 K on a Bruker D8 Venture diffractometer with a Mo Kα (λ = 0.71073 Å) (compounds **3** and **4**) or Cu Kα (λ = 1.54178 Å) (compound **5b**) radiation source. Crystal structures were refined using the SHELXL^62^ software (Table 3). Non-hydrogen atoms were refined anisotropically.

**Table 3:** Crystallographic Data for **3**, **4**, and **5b**

	**3**	**4**	**5b**
chemical formula	C_23_H_18_BrN_4_O_4_Re	C_22_H_15_BrFN_4_O_3_Re	C_26_H_24_BrN_4_O_3_Re
*M*_r_	680.52	668.49	706.6
cryst syst	triclinic	monoclinic	triclinic
color, habit	purple, needle	purple, needle	purple, block
size (nm)	0.42 ×0.13× 0.04	0.70 × 0.170 × 0.060	0.40 × 0.33 × 0.20
space group	*P*1̅ (No.2)	*P*21/*n* (No.14)	*P*1̅ (No.2)
*a* (Å)	9.0463(6)	10.095(2)	9.9235(12)
*b* (Å)	11.0459(7)	18.586(6)	10.9196(13)
c (Å)	12.7033(8)	12.219(4)	13.0134(15)
α (deg)	107.379(2)	90	77.085(4)
β (deg)	94.935(3)	107.191(8)	72.218(4)
γ (deg)	103.227(2)	90	75.163(4)
*V* (Å^3^)	1162.89(13)	2190.1(11)	1281.7(3)
*Z*	2	4	2
ρ_calc_ (g·cm^–3^)	2.302	2.027	1.831
radiation, λ (Å)	Mo, Kα, 0.71073	Mo, Kα, 0.71073	Cu, Kα, 1.54178
μ(Mo, Kα) (mm^–1^)	8.777	7.413	11.384
*F*(000)	750	1272	684
temp (K)	100(2)	100(2)	100(2)
θ range (deg)	3.028–27.191	3.044–27.200	3.612–70.304
data collected (*h*, *k*, *l*)	–11:11, −14:14, −16:16	–12:12, −23:23, −15:15	–12:12, −13:13, −15:15
no. of reflns collected	45,523	27,390	21,942
no. of indep reflns	5166	4818	4526
obsd reflns *F*_o_ ≥ 2.0σ(*F*_o_)	4966	4288	4396
*R*(*F*) [obsd reflns] (%)	1.78	2.81	2.19
*R*_w_(*F*^2^) [all reflns] (%)	5.24	6.13	5.51
GOF	1.051	1.192	1.119
weighting *a*, *b*	0.03300, 1.26140	0.0000, 12.4515	0.0000, 2.9889
params refined	303	293	324
min, max residual densities	–1.04, 1.32	–1.61, 1.82	–0.83, 0.85

### Computational Details

Density functional theory (DFT) calculations were carried out in Gaussian 16 Revision C.02^63^ software and visualized using Gaussview 6^64^ or Avogadro.^65^ Geometry optimizations in the ground state were performed in the gas phase at a MN15L^51^ level of theory combined with a triple ζ-basis set: def2tzvp.^52^ The carbonyl frequency values were scaled using a factor of 0.9578^66^ (see Table S1). TDDFT calculations were performed on the optimized structures at the CAM-B3LYP/def2tzvp level of theory. The solvent effect was simulated using the continuum polarized model (CPM).^67^

### Procedure for the Synthesis of Complexes **1**–**4**

Equimolar amounts of [ReBr(CO)_5_] and the corresponding ligand were poured into a two-necked round bottom flask and dissolved in 20 mL of toluene. The reaction was heated up at reflux for 1 h observing that the mixture darkened upon completion. The solvent was evaporated to dryness. Specific details for the purification of the entitled complexes are mentioned below.

### **1** (C_22_H_16_BrN_4_O_3_Re)

[ReBr(CO)_5_] (0.0934 g, 0.230 mmol), **L1H** (0.06938 g, 0.230 mmol). Work-up: 5 mL of pentane was added to the mixture, and the crude was stirred for 30 min allowing the formation of a dark-crimson solid material. The compound was filtered out and rinsed with pentane (3 × 5 mL). (41.8 mg, 27.9%). ^1^H NMR (CDCl_3_, 25 °C, 400 MHz) δ/ppm: 7.30 (d, 2 H,^3^*J* = 8 Hz, Ph-NH *o*-H), 7.39 (t, 1 H,^3^*J* = 8 Hz, Ph-NH *p*-H), 7.45–7.57 (m, 5H, Ph-NH *m*-H, Ph-N=N *m*-H, Ph-NC *p*-H), 7.64 (m, 3H, Ph-NC *m*-H, Ph-N=N p-H,) 7.84 (d, 2 H,^3^*J* = 8 Hz, Ph-NC *o*-H), 7.89 (d, 2 H,^3^*J* = 8 Hz, Ph-N=N *o*-H), 8.58 (s, 1 H, NH). ^13^C{^1^H} NMR (CDCl_3_, 25 °C, 150 MHz) δ/ppm: 123.21 (Ph-NH *o*-CH), 123.95 (Ph-N=N *o*-CH), 127.90 (Ph-NH *p*-CH), 128.48 (Ph-CN *ipso*-C), 128.93 (Ph-CN *o*-CH), 129.31 (Ph-N=N *m*-CH), 129.39 (Ph-NH *m*-CH), 130.10 (Ph-CN *m*-CH), 131.69 (Ph-N=N *p*-CH), 131.86 (Ph-CN *p*-CH), 140.83 (Ph-NH *ipso*-C), 157.20 (Ph-N=N *ipso*-C), 164.65 (NCN C), 185.33 (CO *trans* Br C), 192.35 (CO *trans* Ph-NH-N C), 192.89 (CO *trans* Ph-N=N C). IR(CH_2_Cl_2_) ν(CO)/cm^–1^: 2035(s), 1959(s), 1923(s). MS (FAB+) (*m*/*z*): [MH + 2]^+^ = 653, [MH]^+^ = 651, [MH-CO]^+^ = 623, [MH-3CO]^+^ = 567. HRMS (ESI +) (*m*/*z*): Calcd. for [MH] ^+^ = 651.004167. Found = 651.00337. [MH-3CO]^+^ = 567.01942. Found = 567.01817.

### **2** (C_23_H_18_BrN_4_O_3_Re)

[ReBr(CO)_5_] (0.1235 g, 0.304 mmol), **L2H** (0.0959 g 0.305 mmol). Work-up: similar to the procedure described above. (93.3 mg, 46.0%). ^1^H NMR (CDCl_3_, 25 °C, 600 MHz) δ/ppm: 2.47 (s, 1 H, CH_3_), 7.29 (d, 2 H,^3^*J* = 8 Hz, Ph-NH *o*-H), 7.38 (t, 1 H,^3^*J* = 7 Hz, Ph-NH *p*-H), 7.43–7.56 (m, 7H, Ph-NH *m*-H, Ph-N=N *m*-H, *p*-tol *m*-H, Ph-N=N *p*-H), 7.74 (d, 2 H,^3^*J* = 8 Hz, *p*-tol *o*-H), 7.88 (d, 2 H,^3^*J* = 7 Hz, Ph-N=N *o*-H), 8.56 (s, 1 H, NH). ^13^C{^1^H} NMR (CDCl_3_, 25 °C, 150 MHz) δ/ppm: 21.71 (CH_3_), 123.11 (Ph-NH *o*-CH), 123.97 (Ph-N=N *o*-CH), 125.58 (*p*-tol *ipso*-C), 127.75 (Ph-NH *p*-CH), 128.85 (*p*-tol *o*-CH), 129.29 (Ph-NH *m*-CH), 129.40 (Ph-N=N *m*-CH), 130.72 (*p*-tol *m*-CH), 131.65 (Ph-N=N *p*-CH), 140.96 (Ph-NH *ipso*-C), 142.49 (*p*-tol *p*-C), 157.24 (Ph-N=N *ipso*-C), 165.02 (NCN C), 185.37 (CO *trans* Br C), 192.40 (CO *trans* Ph-NH-N C), 192.88 (CO *trans* Ph-N=N C). IR(CH_2_Cl_2_) ν(CO)/cm^–1^: 2035(s), 1959(s), 1924(s). MS (DART+) (*m*/*z*): [MH + 2]^+^ = 667, [MH]^+^ = 665, [MH-CO]^+^ = 637, [MH-2CO]^+^ = 609, [MH-3CO]^+^ = 581. HRMS (ESI+) (*m*/*z*): Calcd. for [MH] ^+^ = 665.01982. Found = 665.01917 [MH-3CO]^+^ = 581.035072. Found = 581.03365.

### **3** (C_23_H_18_BrN_4_O_4_Re)

[ReBr(CO)_5_] (0.0930 g, 0.229 mmol), **L3H** (0.0758 g, 0.229 mmol). Work-up: the compound was recrystallized by slow diffusion of 15 mL of pentane into 5 mL of a DCM solution of **3**. The system was kept in the freezer for 1 day allowing the formation of crystalline material. The solid was washed with 3 × 5 mL of pentane. (105 mg, 67.07%). ^1^H NMR (CDCl_3_, 25 °C, 600 MHz) δ/ppm: 3.88 (s, 1 H, CH_3_O H), 7.10 (d, 2 H,^3^*J* = 8 Hz, *p*-CH_3_OPh *m*-H), 7.27 (d, 2 H,^3^*J* = 8 Hz, Ph-NH *o*-H), 7.35 (t, 1 H,^3^*J* = 8 Hz, Ph-NH *p*-H), 7.45 (t, 2H,^3^*J* = 8 Hz, Ph-N=N *m*-H), 7.49 (t, 2H,^3^*J* = 8 Hz, Ph-NH *m*-H), 7.53 (t, 1H,^3^*J* = 7 Hz, Ph-N=N *p*-H), 7.81 (d, 2 H,^3^*J* = 6 Hz, *p*-CH_3_OPh *o*-H), 7.88 (d, 2 H,^3^*J* = 8 Hz, Ph-N=N *o*-H), 8.47 (s, 1 H, NH). ^13^C{^1^H} NMR (CDCl_3_, 25 °C, 150 MHz) δ/ppm: 55.64 (CH_3_O_,_ C), 115.39 (*p*-CH_3_OPh *m*-CH), 120.64 (*p*-CH_3_OPh *ipso*-C), 122.80 (Ph-NH *o*-CH), 123.96 (Ph-N=N *o*-CH), 127.52 (Ph-NH *p*-CH), 129.29 (Ph-NH *m*-CH), 129.41 (Ph-N=N *m*-CH), 130.82 (*p*-CH_3_OPh *o*-CH), 131.68 (Ph-N=N *p*-CH), 141.14 (Ph-NH *ipso*-CH), 157.26 (Ph-N=N *ipso*-C), 162.17 (*p*-CH_3_OPh *p*-C), 165.25 (NCN C), 185.38 (CO *trans* Br C), 192.40 (CO *trans* Ph-NH-N C), 192.92 (CO *trans* Ph-N=C C). IR(CH_2_Cl_2_) ν(CO)/cm^–1^: 2034(s), 1958(m), 1923(s). MS (DART+) (*m*/*z*): [MH + 2]^+^ = 683, [MH]^+^ = 681, [MH-CO]^+^ = 653, [MH-2CO]^+^ = 625, [MH-3CO]^+^ = 597. Anal. Calcd. For (**C_23_H_18_BrN_4_O_4_Re**): C 40.59, H 2.67, N 8.23; found C 39.81, H 2.59, N 7.90.

### **4** (C_22_H_15_BrFN_4_O_3_Re)

[ReBr(CO)_5_] (0.0951 g, 0.234 mmol), **L4H** (0.0745 g, 0.234 mmol). Work-up: after solvent evaporation, the crude was recrystallized by diffusion of pentane into a CHCl_3_ solution at −30 °C. The crystalline material was filtered out and rinsed with 3 × 5 mL of pentane. (96 mg, 61.3%). ^1^H NMR (CDCl_3_, 25 °C, 600 MHz) δ/ppm: 7.32 (d, 2H,^3^*J* = 8 Hz, Ph-NH *o*-H), 7.35 (t, 2H,^3^*J_H-H_* = 8 Hz,^3^*J_H-F_* = 8 Hz, *p*-FPh *m*-H), 7.41 (t, 1H,^3^*J* = 7 Hz, Ph-NH *p*-H), 7.49 (t, 2H, ,^3^*J* = 8 Hz, Ph-NH *m*-H), 7.54 (t, 2H,^3^*J* = 8 Hz, Ph-N=N *m*-H), 7.58 (t, 1H,^3^*J* = 7 Hz, Ph-N=N *p*-H), 7.90 (m, 4H, Ph-N=N *o*-H, *p*-FPh *o*-H), 8.42 (s, 1H, NH). ^19^F NMR (CDCl_3_, 25 °C, 565 MHz) δ/ppm: −105.92 (m, *p*-FPh F). ^13^C{^1^H} NMR (CDCl_3_, 25 °C, 150 MHz) δ/ppm: 117.38 (^2^*J*_C-F_ = 22 Hz , *p*-FPh *m*-CH), 122.88 (Ph-NH *o*-CH), 123.94 (Ph-N=N *o*-CH), 124.74 (^4^*J*_C-F_ = 3 Hz, *p*-FPh *ipso*-C), 127.85 (Ph-NH *p*-CH), 129.36 (Ph-N=N *m*-CH), 129.47 (Ph-NH *m*-CH), 131.51 (^3^*J*_C-F_ = 9 Hz, *p*-FPh *o*-CH), 131.80 (Ph-N=N *p*-CH), 140.92 (Ph-NH *ipso*-C), 157.30 (Ph-N=N *ipso*-C), 164.39 (*J*_C-F_ = 253.5 Hz, *p*-FPh *p*-C), 164.26 (NCN C), 185.25 (CO *trans* Br C), 192.14 (CO *trans* Ph-NH-N C), 192.63 (CO *trans* Ph-N=C C). IR(CH_2_Cl_2_) ν(CO)/cm^–1^: 2036(s), 1961(s), 1925(s). MS (DART +) (*m*/*z*): [MH]^+^ = 669, [M-CO]^+^ = 641, [M-3CO] = 585. Anal. Calcd. For (C_22_H_15_BrFN_4_O_3_Re): C 39.53, H 2.26, N 8.38; found C 39.12, H 2.04, N 8.29.

### **5a** and **5b** (C_26_H_24_BrN_4_O_3_Re)

[ReBr(CO)_5_] (0.0886 g, 0.22 mmol) and **L5H** ( 0.0778 g, 0.022 mmol) were dissolved in 20 mL of toluene and heated in refluxing toluene for 2.5 h. An oily material was afforded after removal of the volatiles; then, the crude was triturated with 5 mL of pentane, yielding a dark solid. (95 mg, 61.5%). ^1^H NMR (CDCl_3_, 25 °C, 600 MHz) δ/ppm: (**5a**) 1.99 (s, 3H, *p*-tol *p*-CH_3_), 2.05 (s, 3H, Mes-N=N *p*-CH_3_), 2.07 (s, 3H, Mes-N=N *o*-CH_3_), 2.81 (s, 3H, Mes-N=N *o*-CH_3_), 6.64 (s, 1H, Mes-N=N *m*-H), 6.71 (s, 1H, Mes-N=N *m*-H), 6.82 (d, 2H, ,^3^*J* = 6 Hz, Ph-NH *o*-H), 6.93 (m, 3H, Ph-NH *p*-H, *p*-tol *m*-H), 7.05 (t, 2H,^3^*J* = 6 Hz, Ph-NH *m*-H), 7.68 (d, 2H,^3^*J* = 6 Hz, *p*-tol *o*-H), 8.22 (s, 1H, NH). **(5b)** 2.05 (s, 3H, Mes-NH *p*-CH_3_), 2.05 (s, 3H, Mes-NH *o*-CH_3_), 2.09 (s, 3H, *p*-tol *p*-CH_3_), 2.41 (s, 3H, Mes-NH *o*-CH_3_), 6.70 (s, 2H, Mes-NH *m-*H), 6.93 (m, 3H, Ph-N=N *p*-H, *m*-H), 6.99 (d, 2H,^3^*J* = 6 Hz, *p*-tol *m*-H), 7.47 (d, 2H,^3^*J* = 6 Hz, *p*-tol *o*-H), 7.61 (s, 1H, NH). 7.85 (m, 2H, Ph-N=N *o*-H). ^13^C{^1^H} NMR (CDCl_3_, 25 °C, 150 MHz) δ/ppm: **(5a)** 17.80 (Mes *o*-CH_3_), 20.42 (Mes *o-*CH_3_), 20.80 (Mes *p-*CH_3_), 21.37 (*p*-tol *p*-CH_3_) , 121.53 (Ph *o*-CH), 126.68(*p*-tol *p*-C), 126.94 (Ph *m*-CH), 128.6 (Mes *m*-C), 129.24 (*p*-tol *o*-CH), 129.47 17 (Mes *o*-C), 130.17 (Mes *m*-C), 130.48 (*p*-tol *m*-CH), 131.17 (Mes *o*-C), 138.18 (Mes *p*-C), 142.20 (Ph *ipso*-C), 142.20 (*p*-tol *ipso*-C) , 155.20 (Mes *ipso*-C), 167.11 (NNCN C), 186.34 (CO *trans* Br C), 192.25 (CO *trans* Mes-N=N C), 193.04 (CO *trans* PhN-NH C). **(5b)** 18.61 (Mes *p*-CH_3_), 19.56 (Mes *o-*CH_3_), 21.14 (Mes *o-*CH_3_)*,* 21.33 (*p*-tol *p*-CH_3_), 124.09 (Ph *o*-CH), 129.14 (*p*-tol *p*-C), 129.43 (*p*-tol *o*-CH), 129.49 (Mes *m*-C), 129.93 (Mes *m*-C), 130.94 (*p*-tol *m*-CH), 131.23 (Ph *m*-CH), 136.53 (Mes *ipso*-C), 137.70 (Mes *o*-C), 137.89 (Mes *o*-C), 140.68 (Mes *p*-C), 141.79 (*p*-tol *ipso*-C), 157.15 (Ph *ipso*-C), 161.04 (NNCN C), 184.77 (CO *trans* Br C), 192.29 (CO *trans* Mes-NH C), 194.16 (CO *trans* Ph-N=N C). IR(CH_2_Cl_2_) ν(CO)/cm^–1^: 2036(s), 1959(s), 1922(s). MS (DART+) (*m*/*z*): [MH + 2]^+^ = 709, [MH]^+^ = 707, [MH-CO]^+^ = 678, [MH-3CO]^+^ = 623. HRMS (ESI+) (*m*/*z*): Calcd. for [MH] ^+^ = 707.06677. Found = 707.06667. [MH-2CO]^+^ = 651.07684. Found = 651.07610. [MH-3CO]^+^ = 623.08193. Found = 623.08092.

## Supporting Information Available

1D (^1^H, ^13^C{^1^H}) and 2D (^1^H EXSY) NMR experiments, additional electronic spectroscopy measurements, and computational data (geometry optimizations, TDDFT calculations, and NTO analysis) (PDF)
