# Supplementary material for: Neutral Formazan Ligands Bound to the fac-(CO)3Re(I) Fragment: Structural, Spectroscopic, and Computational Studies
Source: Inorg Chem. 2022 Aug 15;61(34):13532–42. doi: 10.1021/acs.inorgchem.2c02168 (PMC9438031; doi:10.1021/acs.inorgchem.2c02168)
Supplement: Supplementary file 1 — ic2c02168_si_001.pdf [file ic2c02168_si_001.pdf]

Supporting information

Neutral Formazan Ligands Bound to the *fac*-$(CO)_3Re(I)$ Fragment: Structural, Spectroscopic and Computational Studies

Liliana Capulín Flores,[a,b] Lucas A. Paul,[c] Inke Siewert,[c] Remco Havenith,[a] Noé Zúñiga-Villarreal,[b] and Edwin Otten[a*]*

[a] Stratingh Institute for Chemistry, University of Groningen, Nijenborgh 4, 9747 AG Groningen, The Netherlands.

[b] Instituto de Química, Universidad Nacional Autónoma de México, Ciudad Universitaria, Circuito Exterior, 04510 México, D.F., México.

[c] Universität Göttingen, Institut für Anorganische Chemie, Tammannstraße 4, D-37077 Göttingen, Germany.

Corresponding authors:

*zuniga@unam.mx

*edwin.otten@rug.nl

1. NMR spectra……………………………………………………………………S2

2. 1H EXSY experiments…………………………………………………………..S8

3. H/D experiments………………………………………………………………S10

4. Computational studies…………………………………………………………..S11

5. Luminescence studies…………………………………………………………S28

- **NMR spectra**

a) ^{1}H

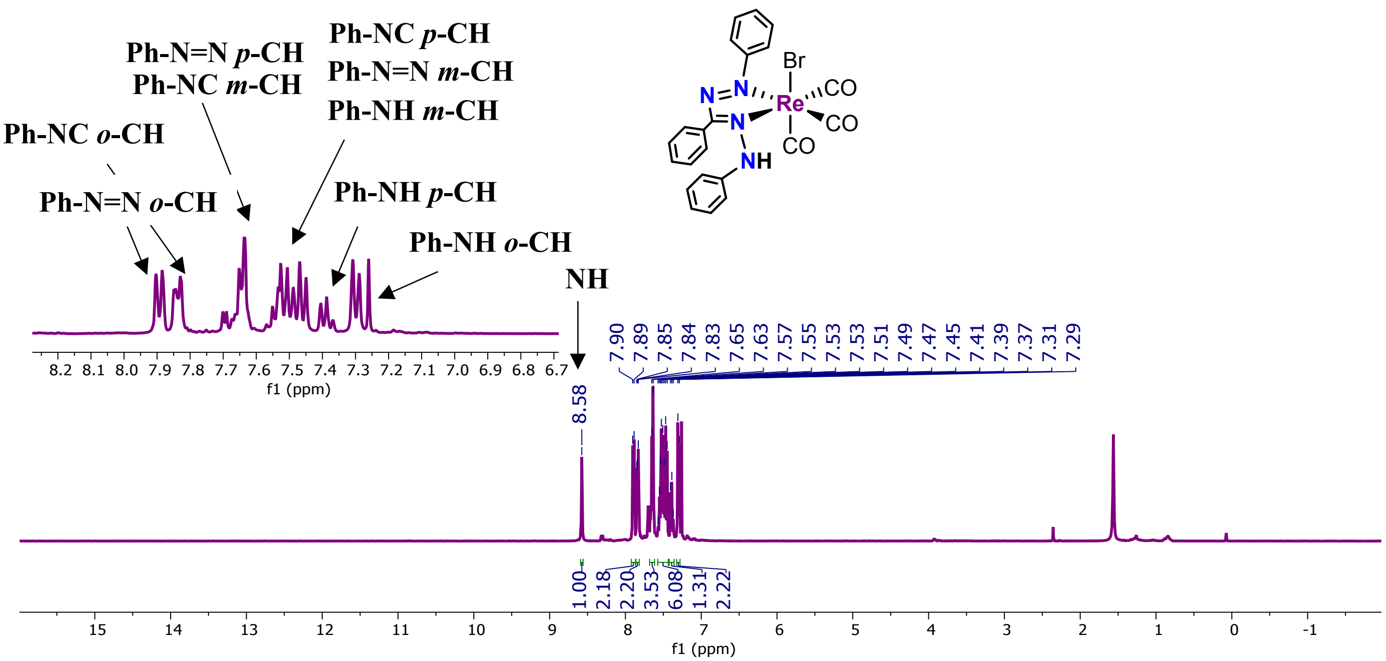

b) $^{13}C\{^{1}H\}$

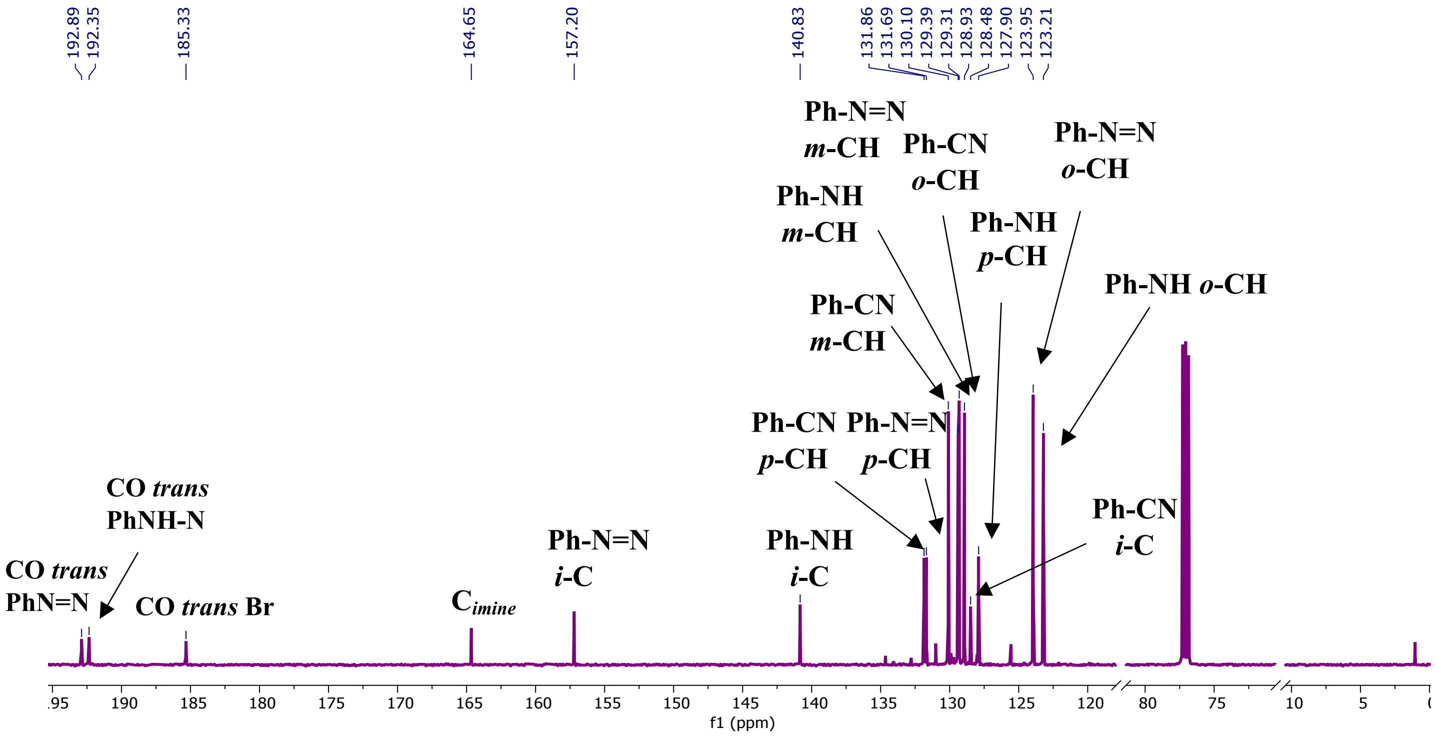

Figure S1. a) ^{1}H and b) $^{13}C\{^{1}H\}$ NMR spectra of 1 in $CDCl_3$.

a) ^{1}H

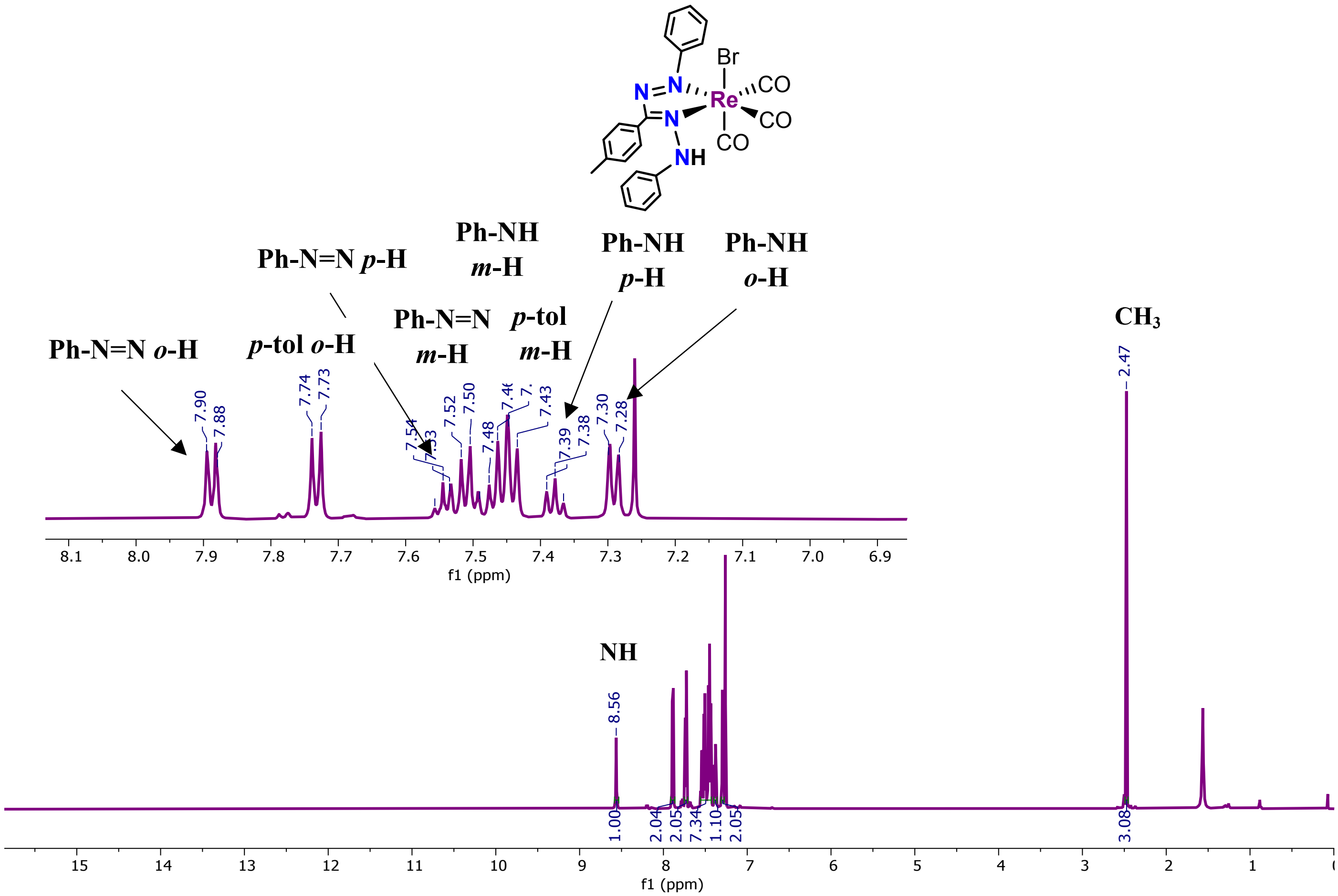

b) $^{13}C\{^{1}H\}$

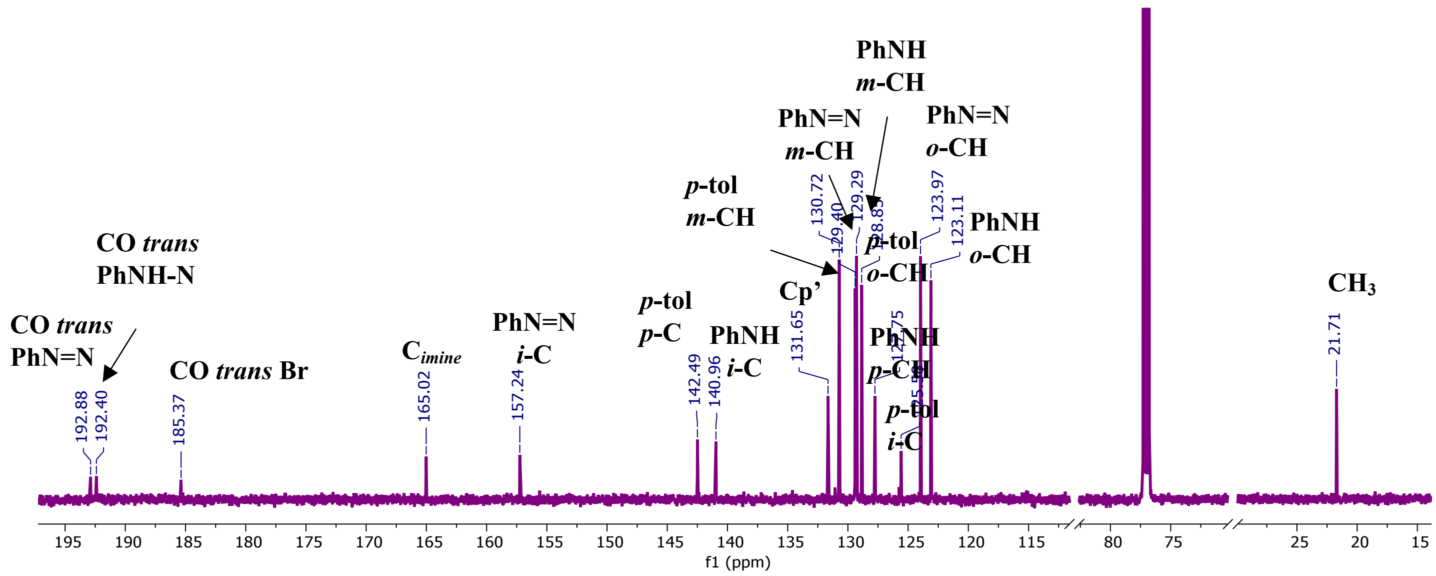

Figure S2. a) ^{1}H and b) $^{13}C\{^{1}H\}$ NMR spectra of 2 in $CDCl_3$.

a)

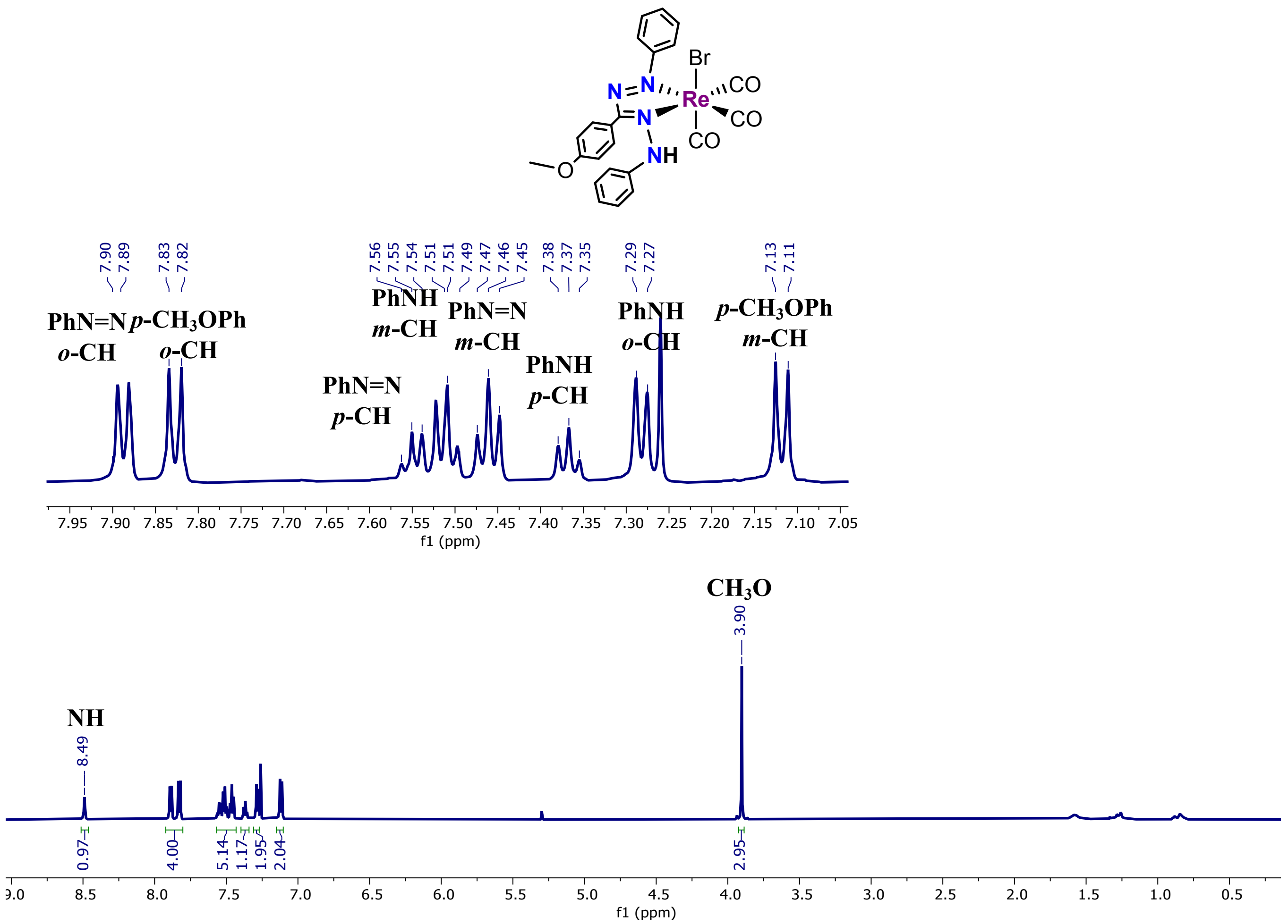

b)

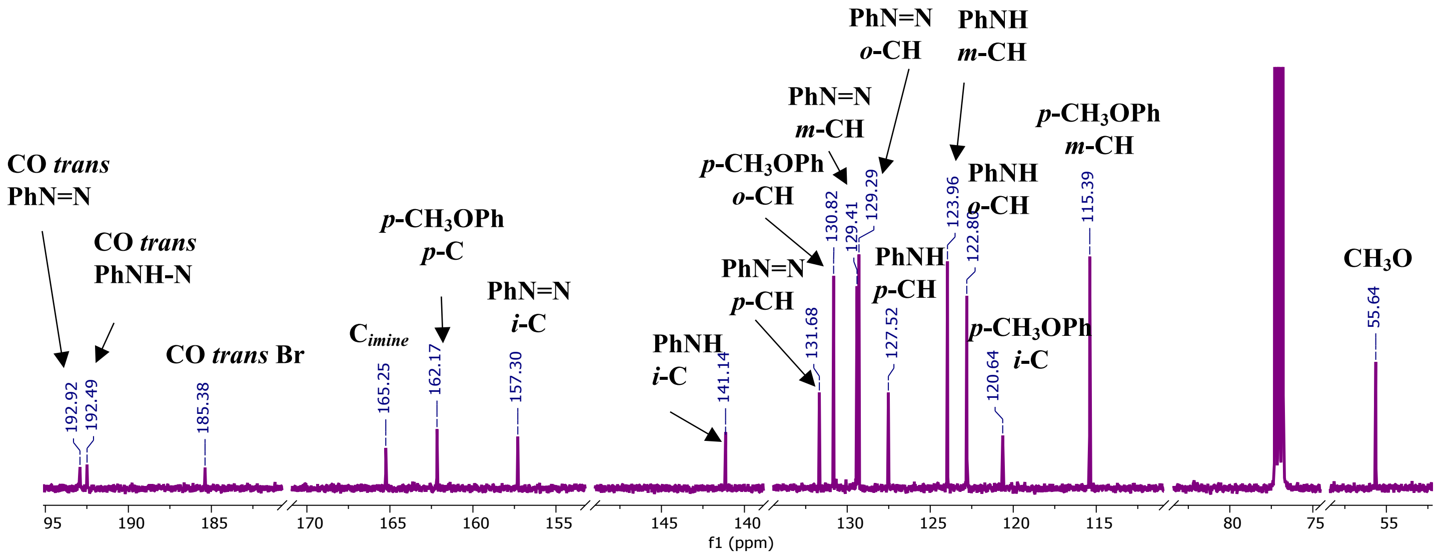

Figure S3. a) ^{1}H and b) $^{13}C\{^1H\}$ NMR spectra of 3 in $CDCl_3$.

a) ^{1}H

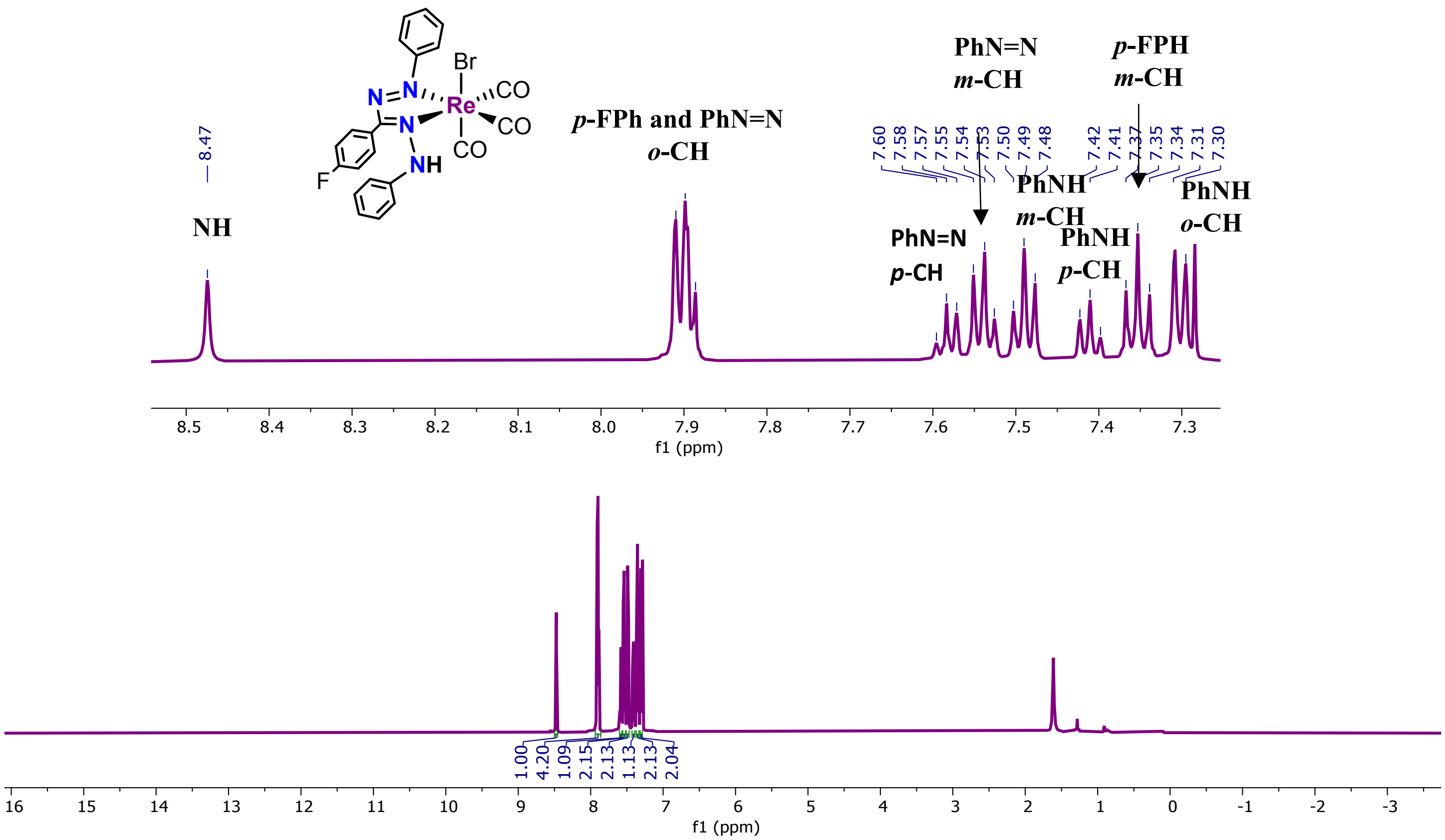

b) $^{13}C\{^{1}H\}$

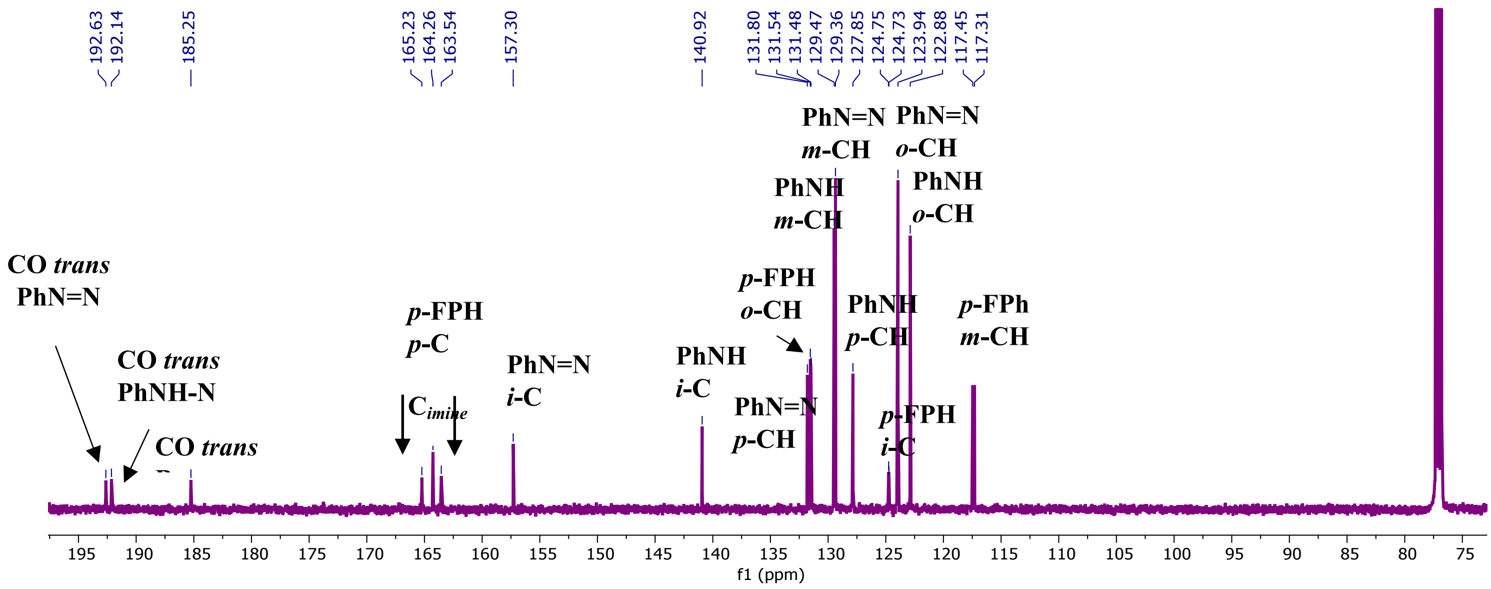

Figure S4. a) ^{1}H and b) $^{13}C\{^{1}H\}$ NMR spectra of 4 in $CDCl_3$.

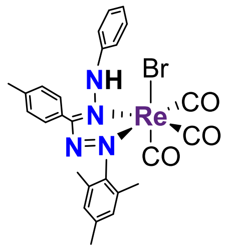

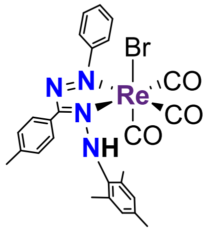

5a **5b**

^{1}H

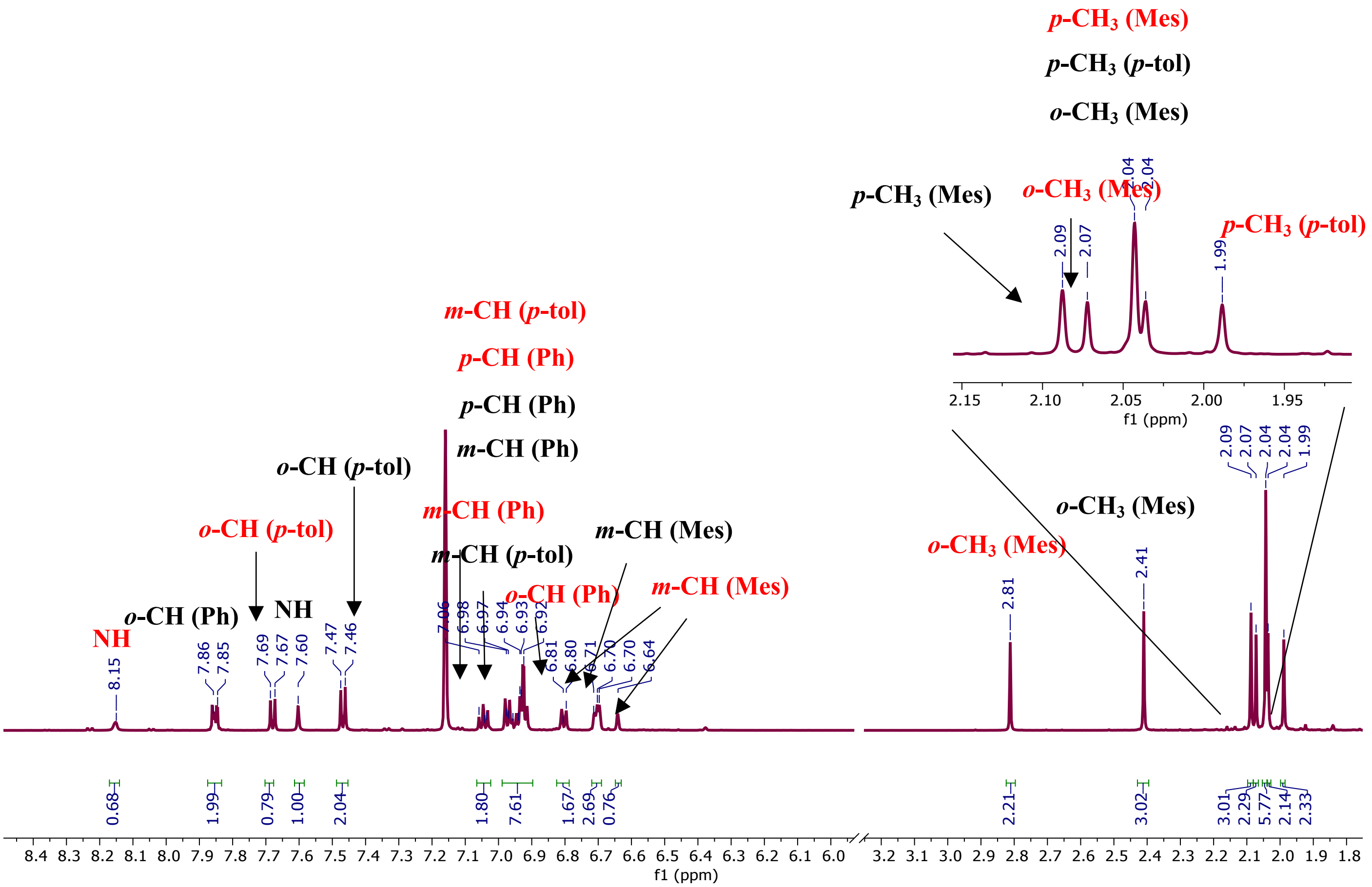

Figure S5. **^{1}H spectrum of the mixture 5a-5b in C_6D_6.**

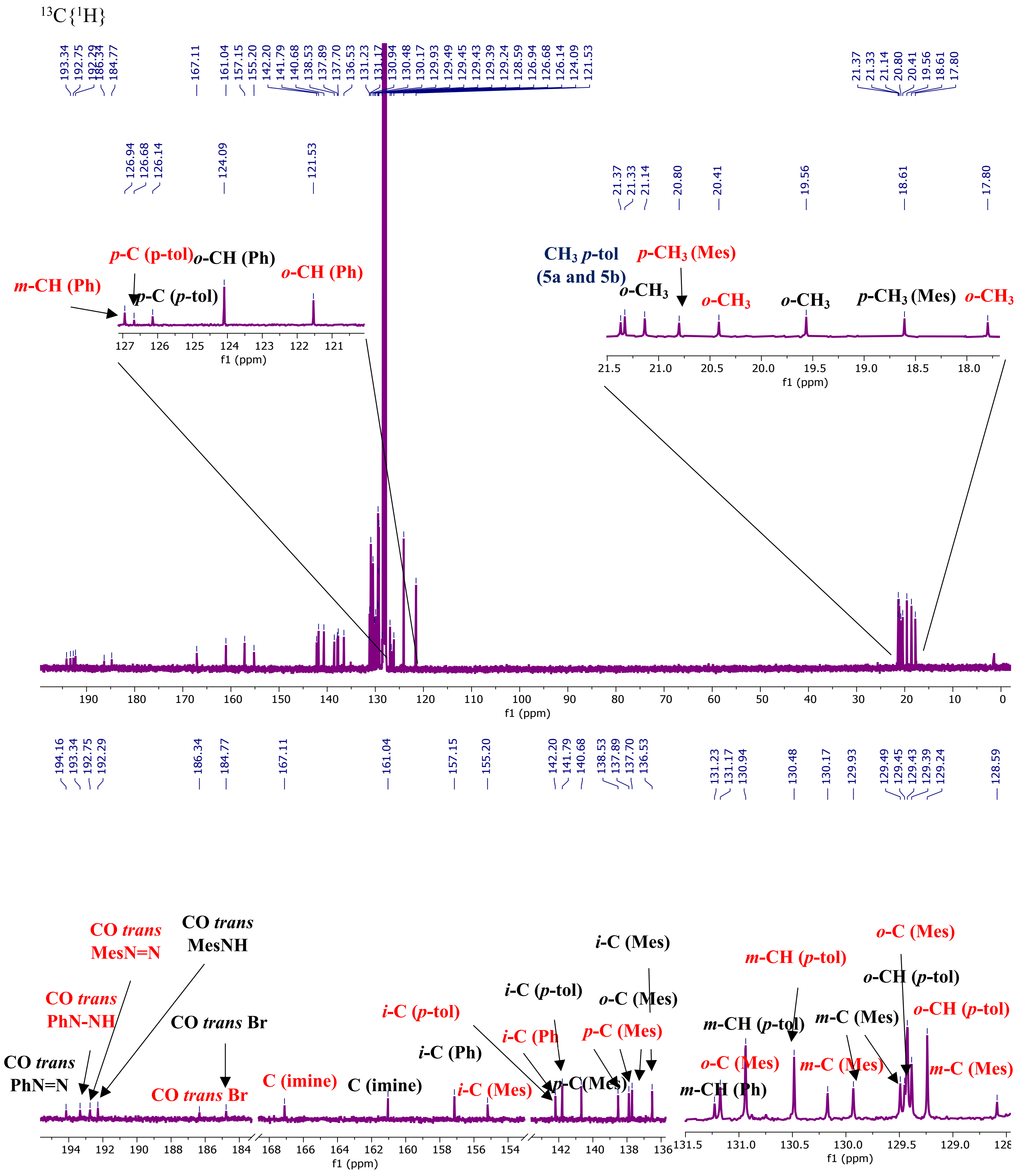

Figure S6. $^{13}C\{^1H\}$ spectrum of the mixture 5a-5b in C_6D_6.

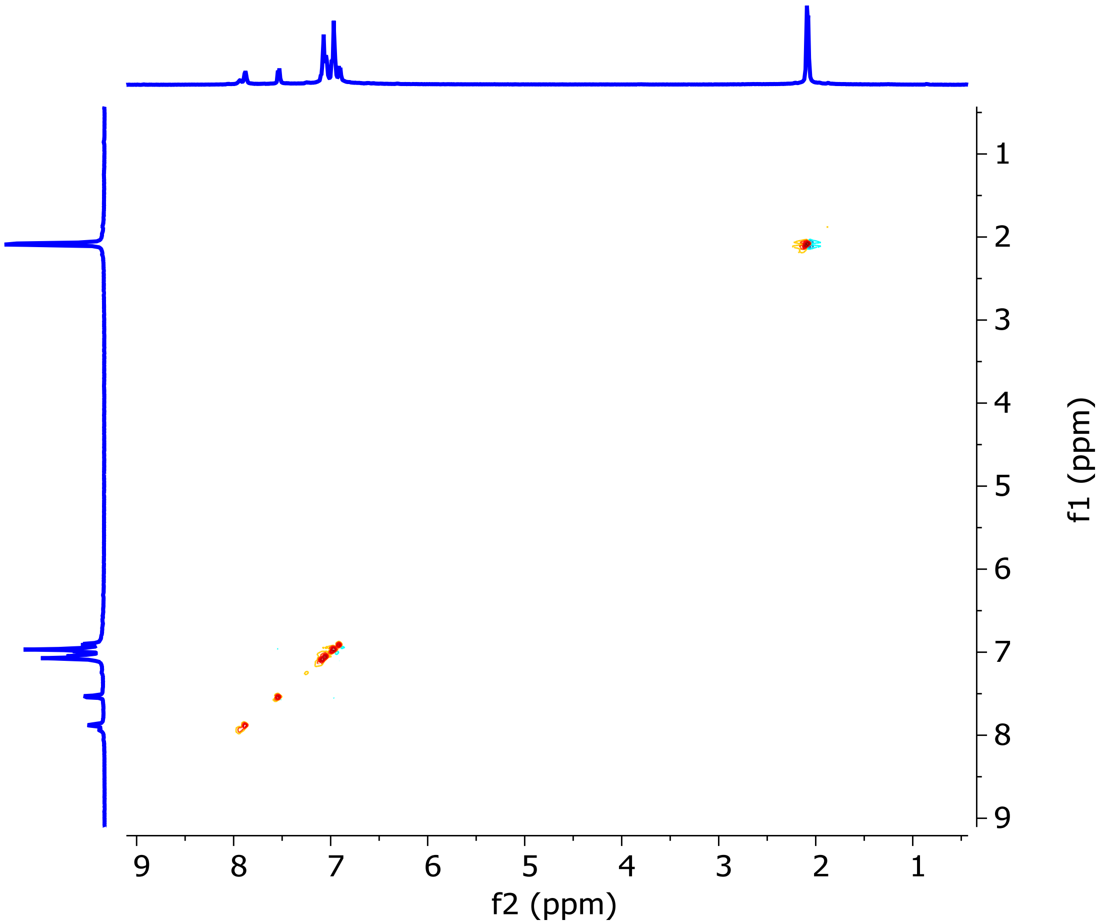

Figure S7. **^{1}H EXSY experiment of 2 at 80°C in C_6D_6.**

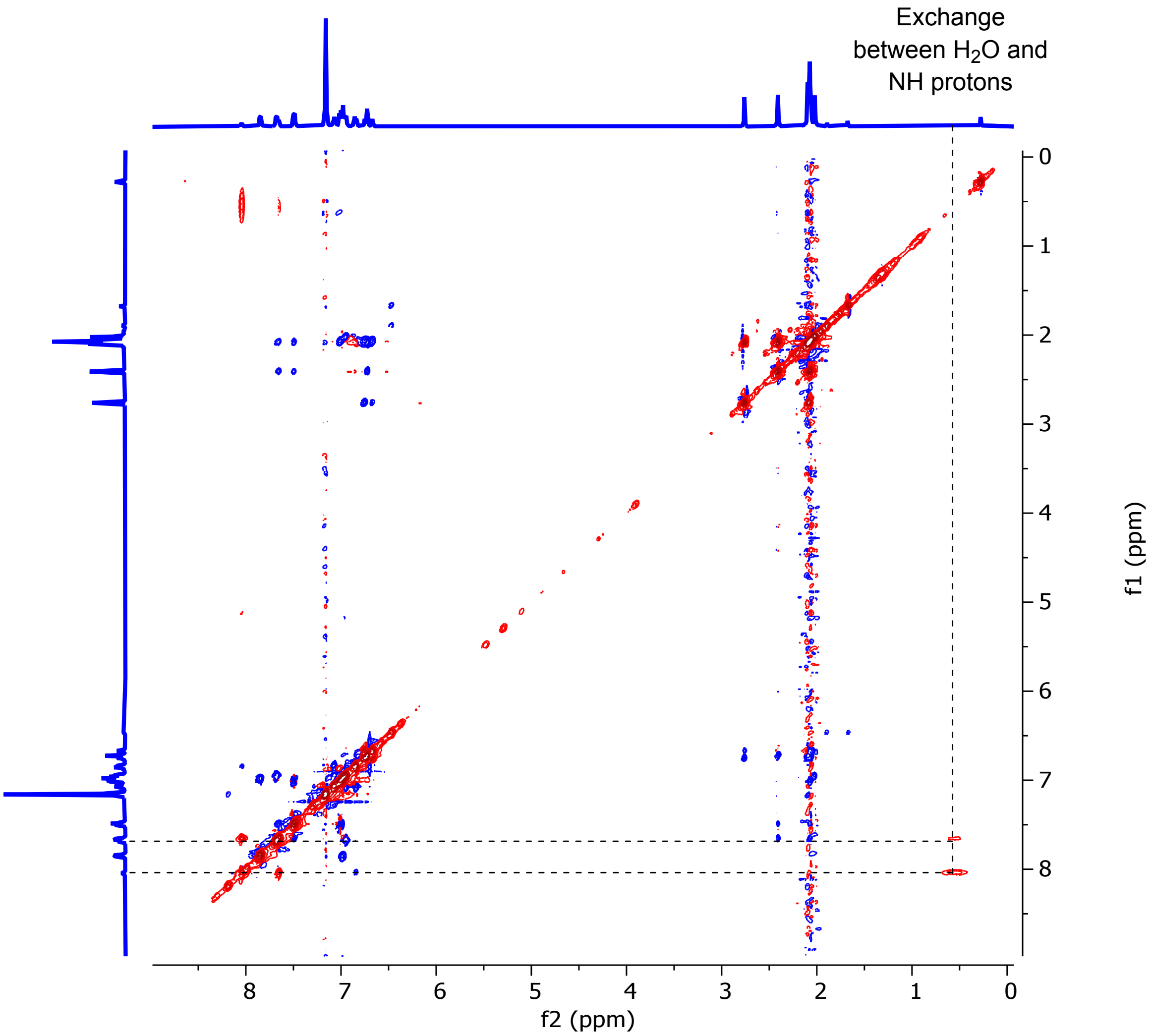

Figure S8. ^1H **EXSY experiment of the mixture 5a/b at 80°C in** C_6D_6 **in the presence of traces of water.**

a) ^{1}H NMR spectrum of a mixture of **4** and its deuterated form **4D** in benzene-d_6 at 25°C

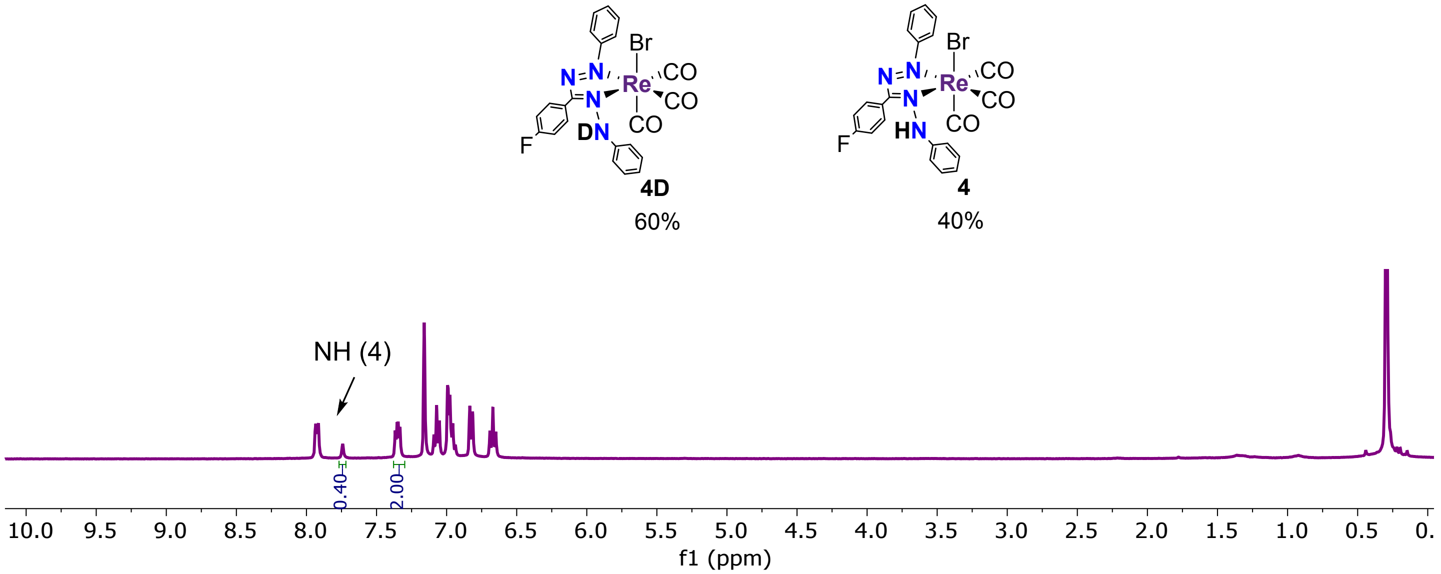

b) ^{1}H NMR spectrum after the additon of **2** (benzene-d_6 at 25°C).

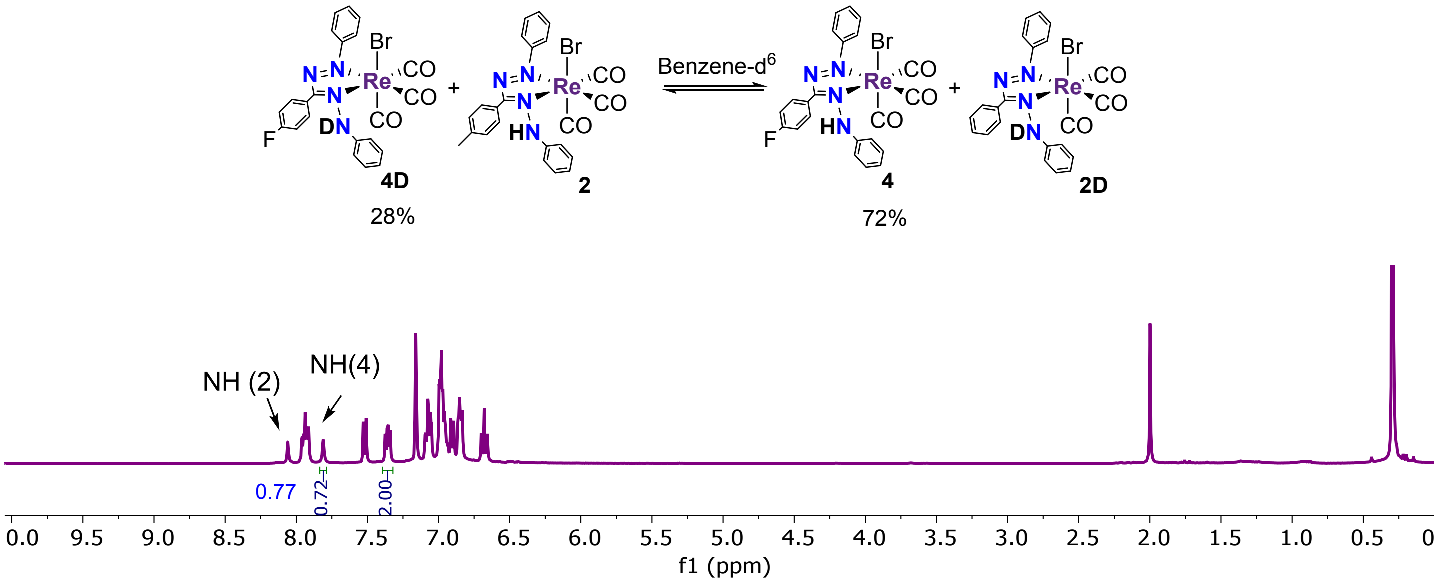

Figure S9. ^{1}H NMR spectra of H/D exchange reactions: a) in compound 4; and b) between compounds 2 and 4 in C_6D_6.

- **Computational studies**

Table S1. Comparison between experimental and theoretical ν(CO) frequencies (scaled by a factor[1] of 0.9578)

Compound ν(CO)/ cm^{-1}					
**4**		**5a**		**5b**	
Experimental	Theoretical	Experimental	Theoretical	Experimental	Theoretical
2036<br>1961<br>1925	2025<br>1965<br>1930	2036<br>1959<br>1922	2026<br>1965<br>1933	2036<br>1959<br>1922	2021<br>1960<br>1926

Table S2. Comparison between the bond-lengths obtained from X-ray diffraction vs theoretical values for 4.

Bond length	Experimental (Å)	Theoretical (Å)	Difference (Å)[a]
Re1-Br1	2.6236(7)	2.65547	**0.03187**
Re1-N1	2.099(6)	2.10272	0.00372
Re1-N3	2.185(5)	2.19551	0.01051
Re1-C20	1.955(6)	1.97518	**0.02018**
Re1-C21	1.919(5)	1.92875	0.00975
Re1-C22	1.918(6)	1.92473	0.00673
N1-N2	1.291(7)	1.2997	0.0087
C7-N2	1.382(7)	1.3701	0.0119
C7-N3	1.319(8)	1.32976	0.01076
N3-N4	1.324(8)	1.37402	**0.05002**

[a]Values in bold indicate that difference is statistically significant. Underestimated bond lengths are reported as negative values.

Table S3. Comparison between the bond-lengths obtained from X-ray diffraction vs theoretical values for 5b.

Bond length	Experimental (Å)	Theoretical (Å)	Difference (Å)[a]
Re1-Br1	2.5946(6)	2.64731	**0.05271**
Re1-N1	2.122(3)	2.11241	**-0.00959**
Re1-N3	2.174(4)	2.18773	**0.01373**
Re1-C20	1.957(5)	1.96716	0.01016
Re1-C21	1.916(6)	1.93083	0.01483
Re1-C22	1.964(4)	1.92394	**-0.04006**
N1-N2	1.293(5)	1.29652	0.00352
C7-N2	1.364(8)	1.3711	0.0071
C7-N3	1.326(5)	1.32938	0.00338
N3-N4	1.343(7)	1.37085	**0.02785**

[a]Values in bold indicate that difference is statistically significant. Underestimated bond lengths are reported as negative values.

- **Compound 4**

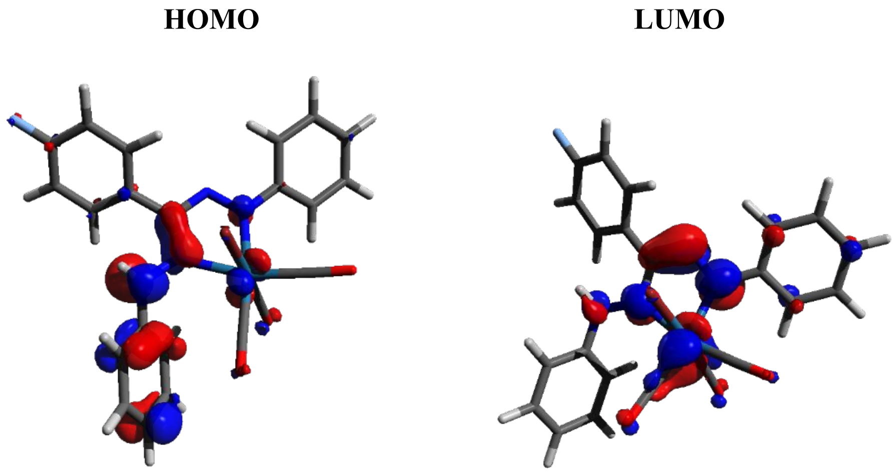

Figure S10. Frontier orbitals for complex 4 plotted with an isovalue of 0.05 from TDDFT calculations (CAM-B3LYP/def2tzvp).

a)

b)

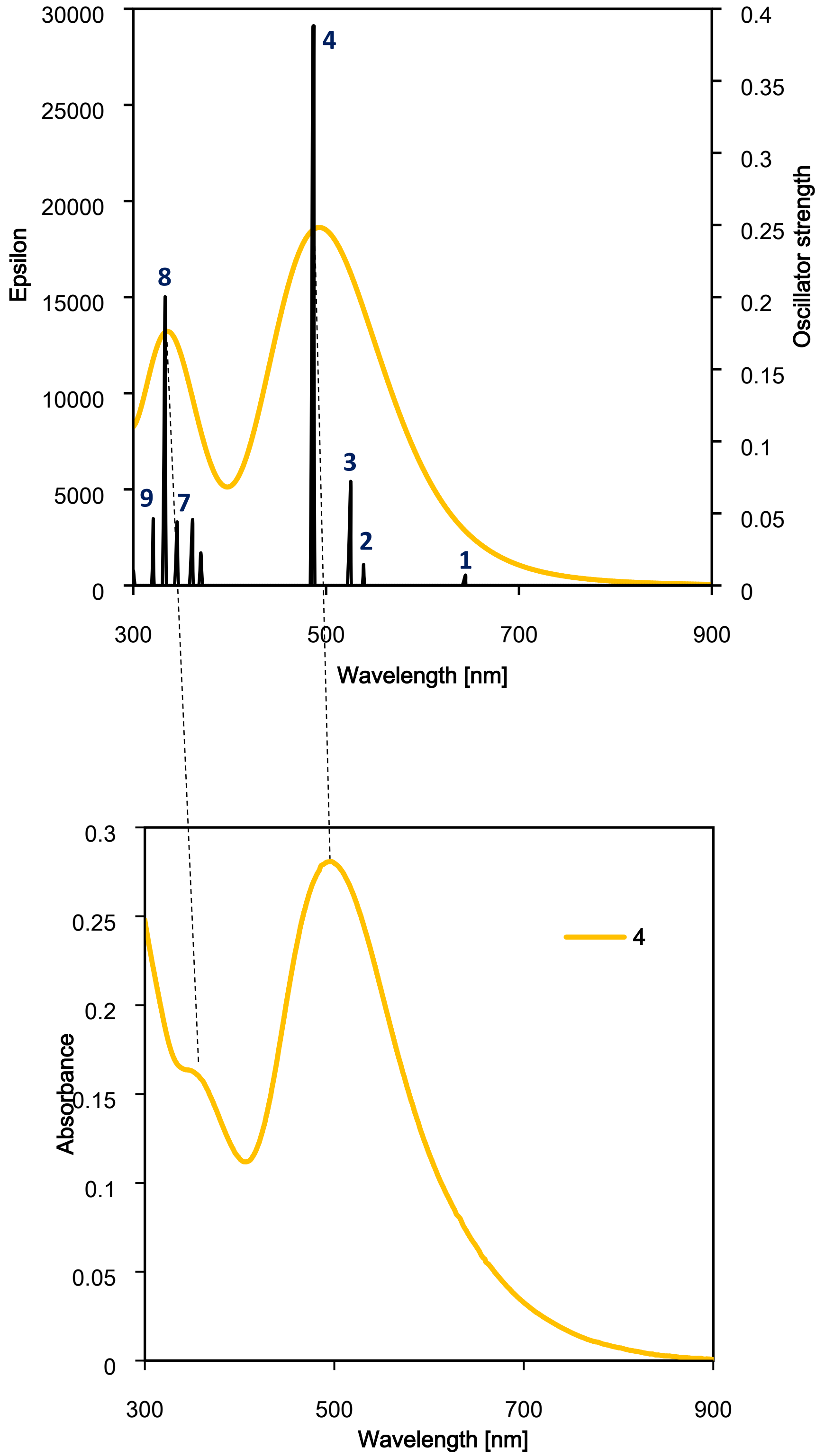

Figure S11. a) Calculated UV-vis for 4 in toluene at CAM-B3LYP/def2tzvp/ CPCM and b) Experimental spectrum in toluene.

Table S4. Natural transitions orbitals (isovalue 0.05) for the main electronic transitions in 4 obtained from TDDFT calculations at CAM-B3LYP/deftzvp, CPCM (toluene) as solvation model

TD-DFT					NTO	
**Excited state**	**Electronic transitions**	**λ_{theo}(nm)/ Energy (eV)**	**λ_{exp}(nm)**	**Oscillator strength**	**Hole → Electron**	**Occupation number**
1	H-1→L<br>H→ L	644.54/1.9236		0.0072	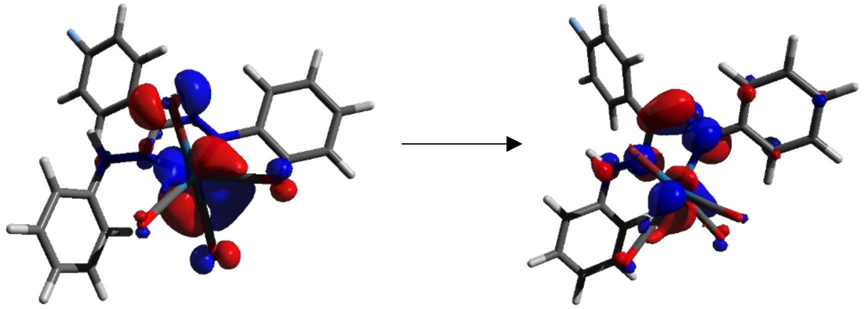	0.99892
2	H-3→L<br>H-2→L<br>H-1→ L<br>H→ L	538.83/2.3010	495 nm	0.0145	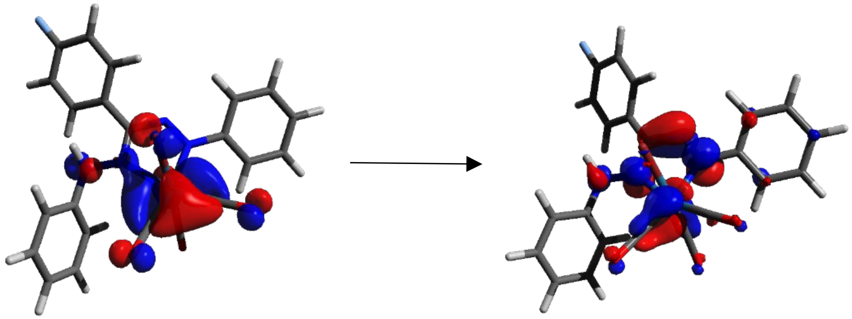	0.99618
3	H-3→ L<br>H-2→L<br>H-1→L<br>H→L	525.58/2.3590		0.0722	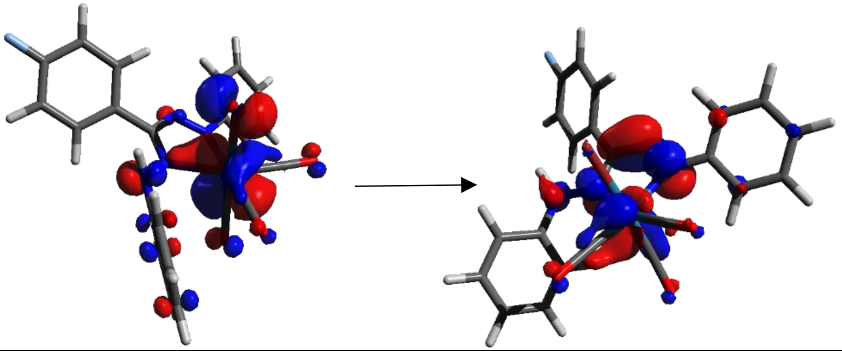	0.99368

4	H-2→ L<br>H-1→ L<br>H → L	487.04/2.5457		0.3878	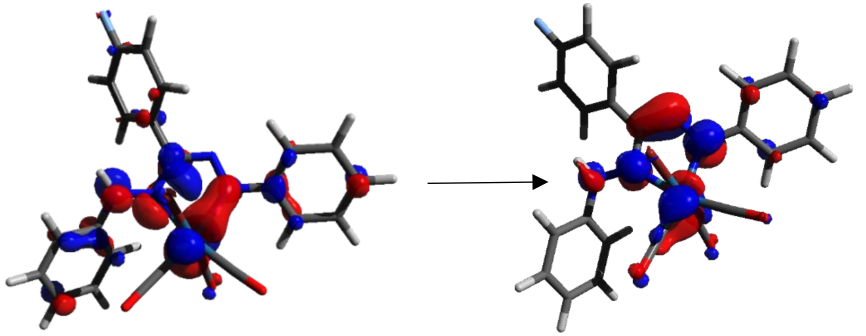	0.98807
7	H-12→L<br>H-9→L<br>H-3→L<br>H-6→L<br>H-5→L<br>H-4→L	345.57/3.5878		0.0441	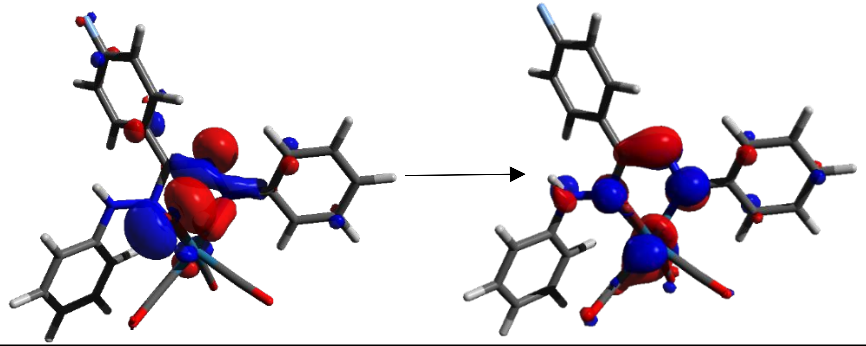	0.98121
8	H-12→L<br>H-10→L<br>H-9→L<br>H-8→L<br>H-6→L<br>H-5→L<br>H-4→L	333.14/3.7217	353	0.2003	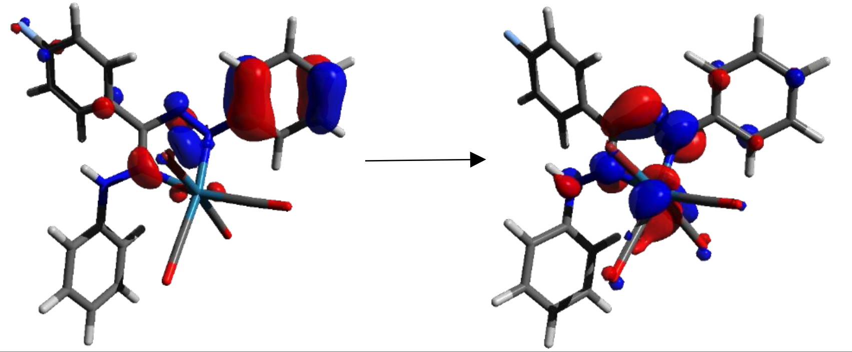	0.97748

9	H-12$\rightarrow$L<br>H-8$\rightarrow$L<br>H-7$\rightarrow$L<br>H-6$\rightarrow$L<br>H-5$\rightarrow$L	320.80/3.8648		0.0463	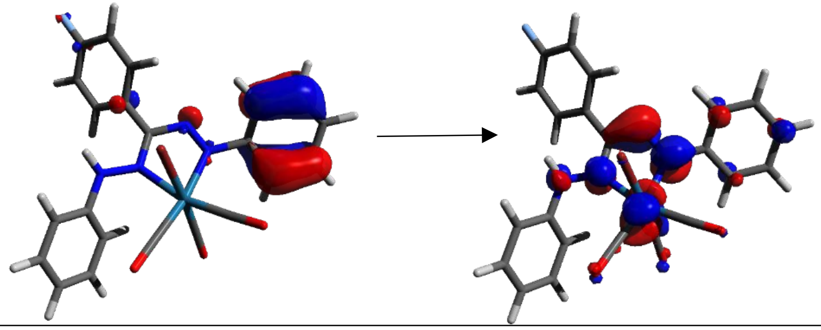	0.97670

Compound 5a

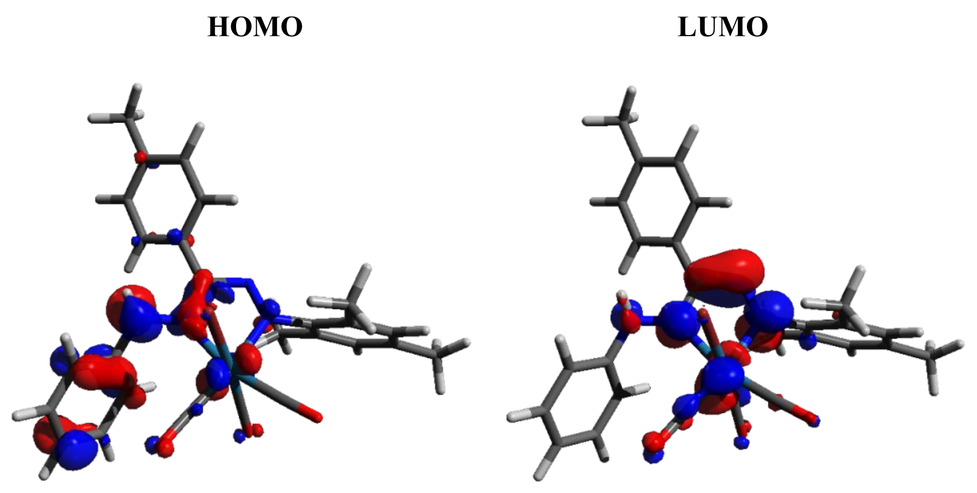

Figure S12. Frontier orbitals for complex 5a plotted with an isovalue of 0.05 from TDDFT calculations (CAM-B3LYP/def2tzvp).

.

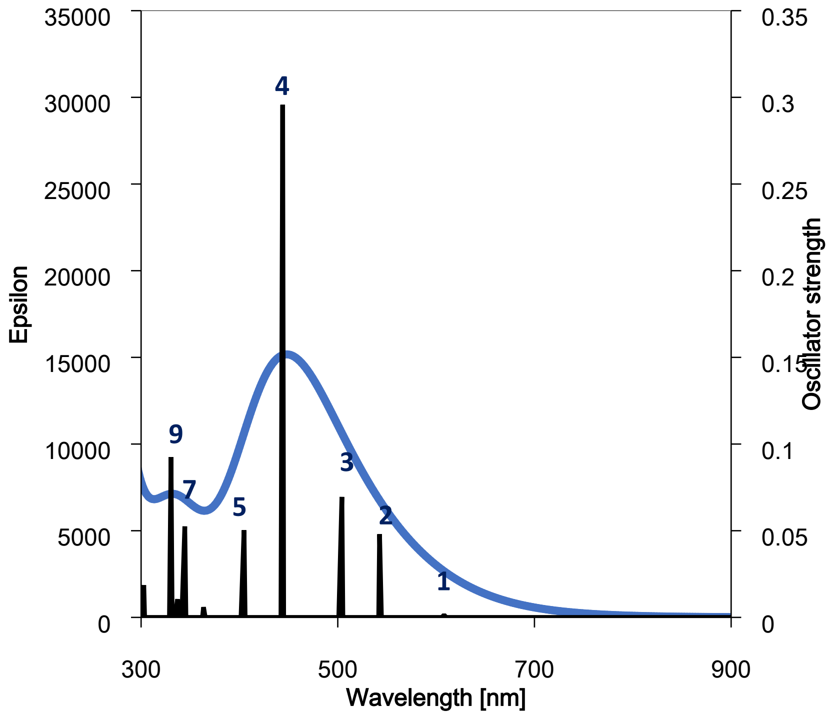

Figure S13. Calculated UV-vis for 5a in toluene at CAM-B3LYP/def2tzvp/CPCM.

Table S5. Natural transitions orbitals (isovalue 0.05) for the main electronic transitions in 5a obtained from TDDFT calculations at CAM-B3LYP/deftzvp, CPCM (toluene) as solvation model.

TD-DFT					NTO	
**Excited state**	**Electronic transitions**	**λ_{theo}(nm)/ Energy (eV)**	**λ_{exp}(nm)/**	**Oscillator strength**	**Hole →Electron**	**Occupation number**
1	H-9→L<br>H-2→<br>H-1→L<br>H→L	608.08/ 2.0389		0.0014	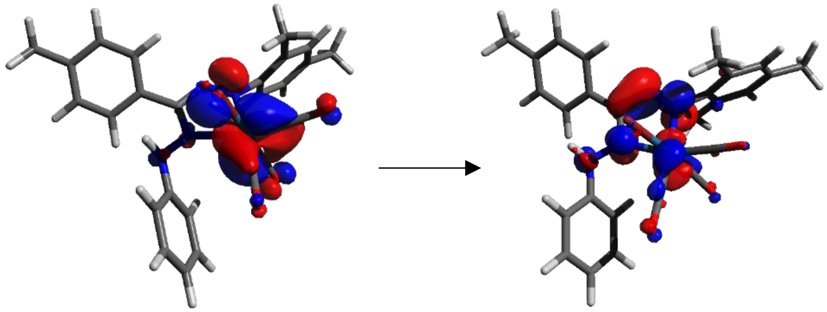	0.99761
2	H-7→L<br>H-5→L<br>H-3→L<br>H-2→ L<br>H-1→ L<br>H→L	542.51/2.2854	520	0.0480	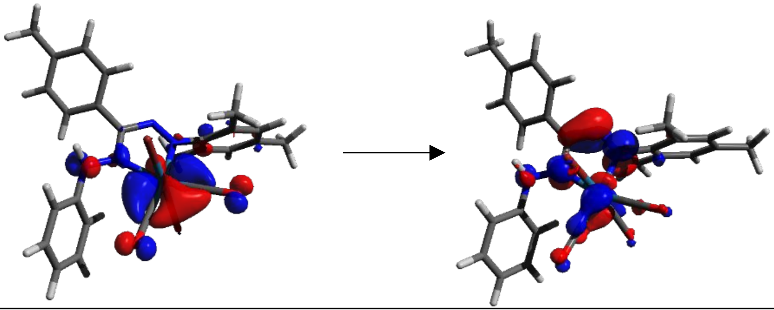	0.99508
3	H-2→L<br>H → L	504.19/2.4591		0.0695	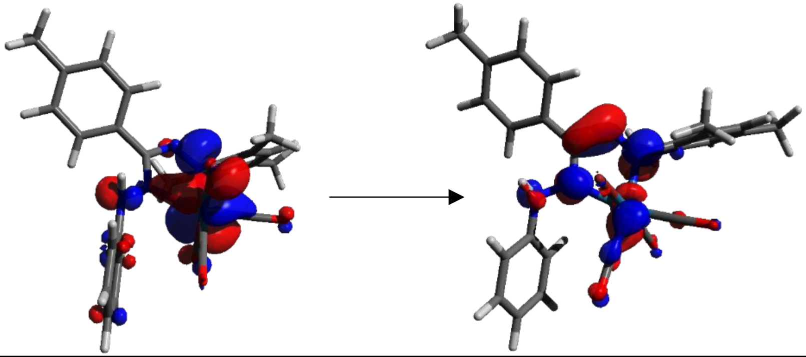	0.99011

4	H-5→L<br>H-3→ L<br>H-2→ L<br>H -1→ L<br>H→L	443.98/2.7925		0.2957	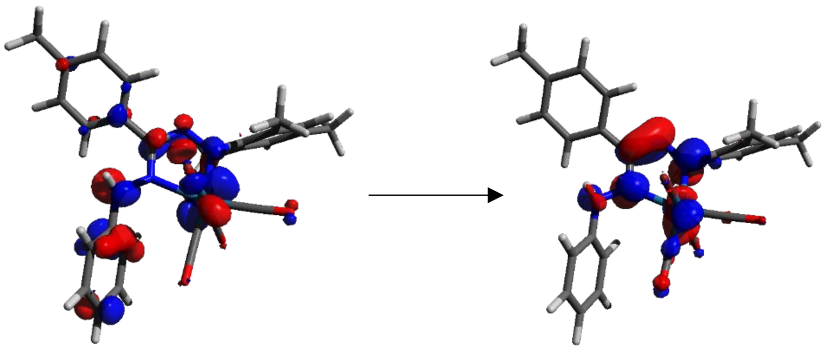	0.98790
5	H-13→L<br>H-12→L<br>H-5→L<br>H-3→L	404.68/3.0638	-----------	0.0503	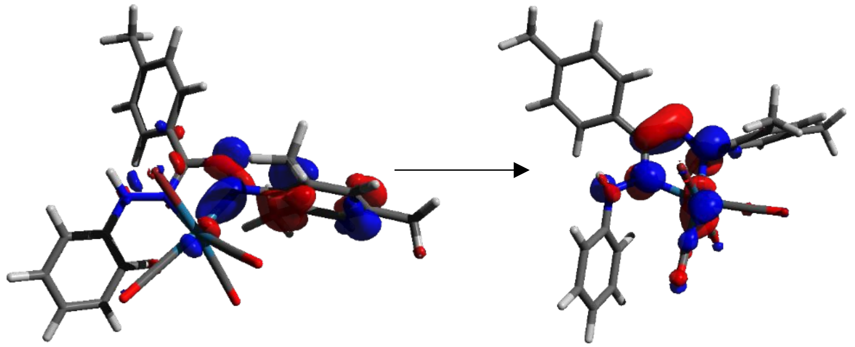	0.98907
7	H-10 → L<br>H-9 → L<br>H-8 → L<br>H-7 → L<br>H-6 → L<br>H-5 → L	344.44/ 3.5996		0.0525	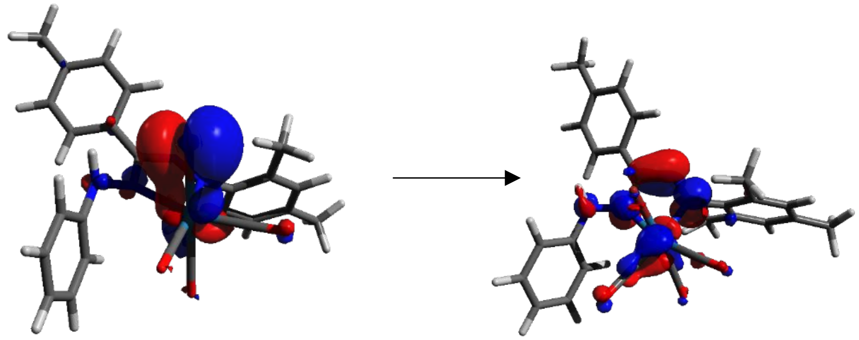	0.98683

9	H-13 $\rightarrow$L<br>H-12$\rightarrow$L<br>H-10 $\rightarrow$L<br>H-9 $\rightarrow$L<br>H-6$\rightarrow$ L	330.43/3.7522		0.0925	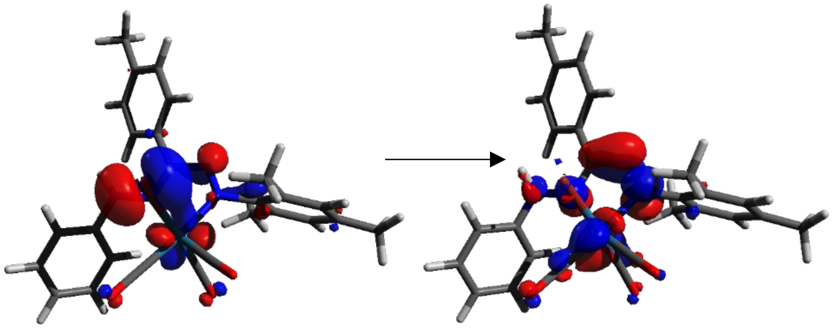	0.97532

Compound 5b

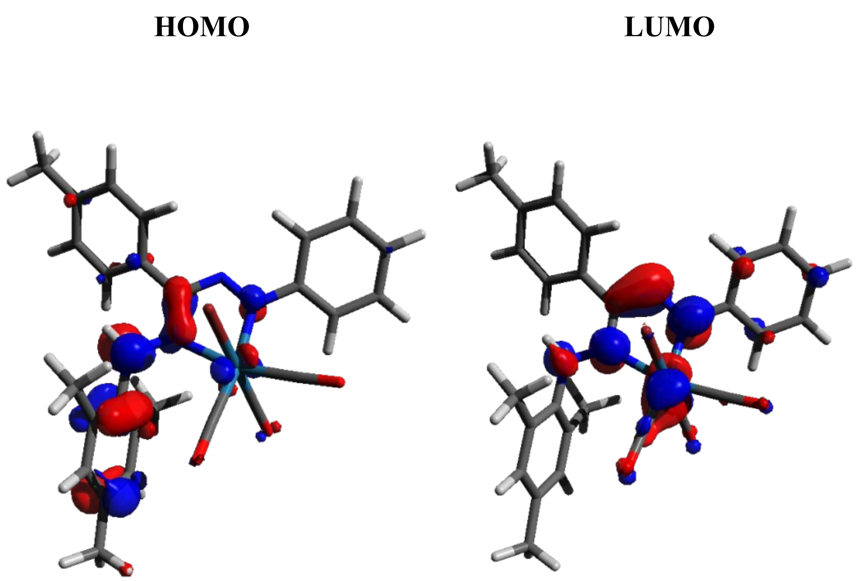

Figure S14. Frontier orbitals for complex 5 plotted with an isovalue of 0.05 from TDDFT calculations (CAM-B3LYP/def2tzvp).

.

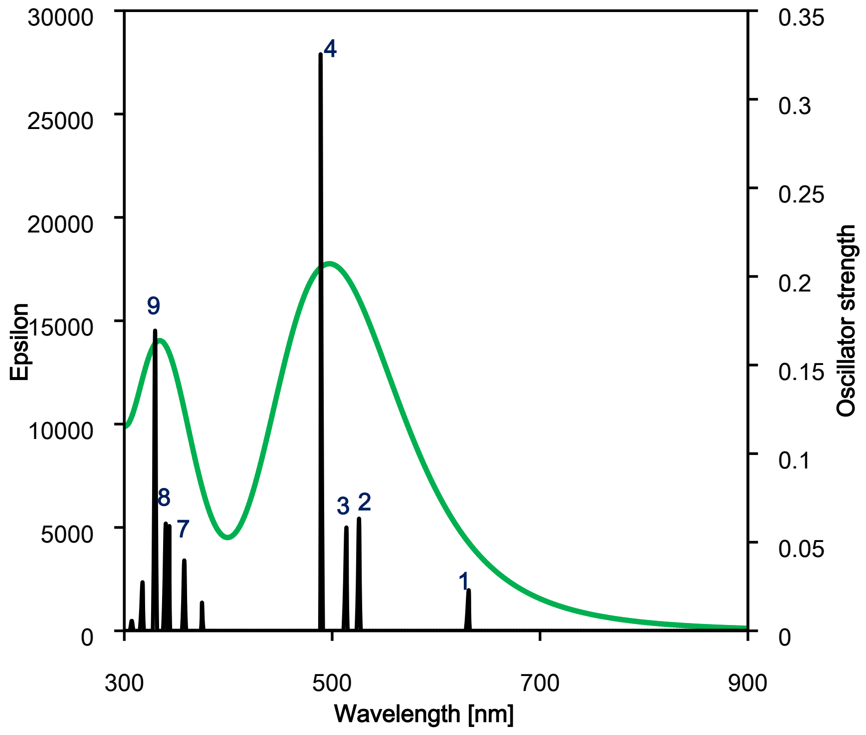

Figure S15. Calculated UV-vis for 5b in toluene at CAM-B3LYP/deftzvp CPCM (toluene).

Table S6. Natural transitions orbitals (isovalue 0.05) for the main electronic transitions in 5b obtained from TDDFT calculations at CAM-B3LYP/deftzvp, CPCM (toluene) as solvation model.

TD-DFT					NTO	
**Excited state**	**Electronic transitions**	**λ_{theo}(nm)/ Energy (eV)**	**λ_{exp}(nm)/**	**Oscillator strength**	**Hole →Electron**	**Occupation Number**
1	H-1→L<br>H→ L	631.38/<br>1.9637		0.0229	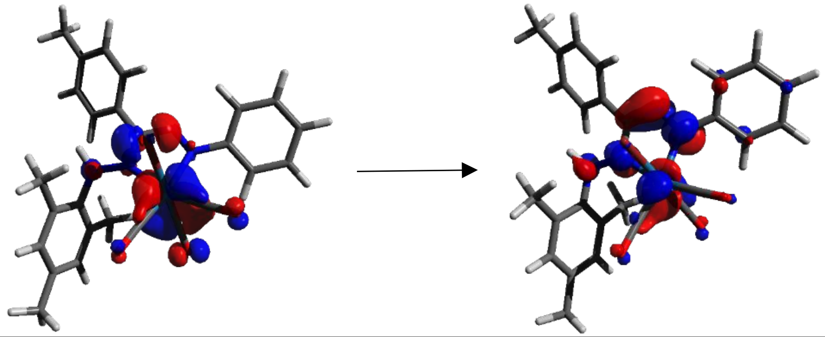	0.99873
2	H-4→L<br>H-3→ L<br>H-2→ L<br>H-1 → L<br>H → L	525.81/2.6393	452	0.0633	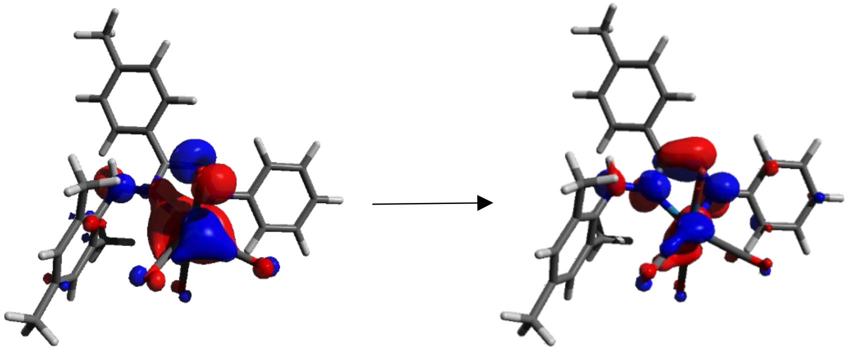	0.99201
3	H-6→ L<br>H-5 → L<br>H-4 → L<br>H-3→L<br>H-1 → L<br>H → L	513.70/2.4136		0.0583	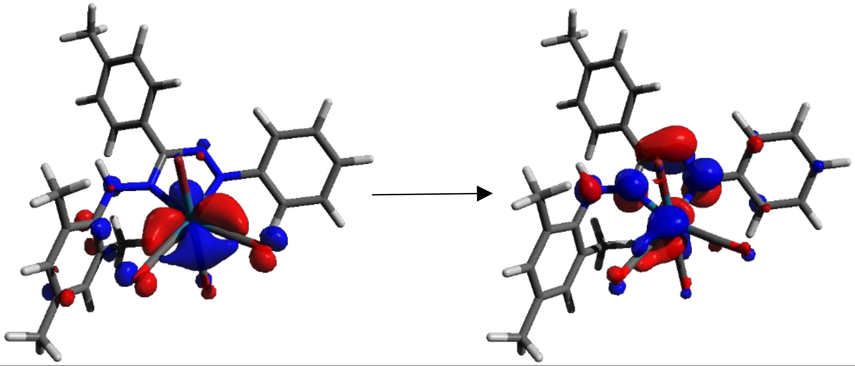	0.99712

4	H-3 → L<br>H-2 → L<br>H-1 → L<br>H → L	488.84/2.5363		0.3254	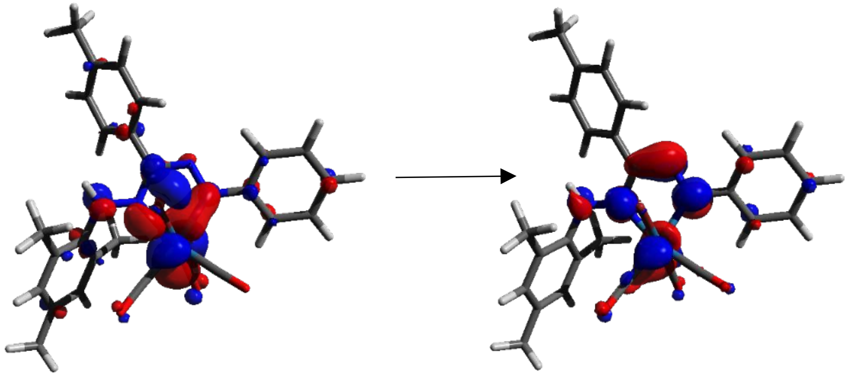	0.98823
7	H-13 →L<br>H-10 →L<br>H-9 →L<br>H-6 → L<br>H-4 →L<br>H-3 →L	343.37/3.6108	-------	0.0591	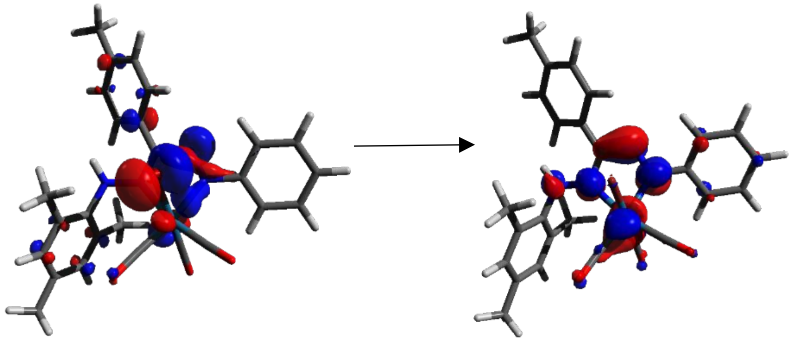	0.98084
8	H-13 → L<br>H-6 → L<br>H-5 → L<br>H-4 → L<br>H-3 → L	339.90/3.6477		0.0605	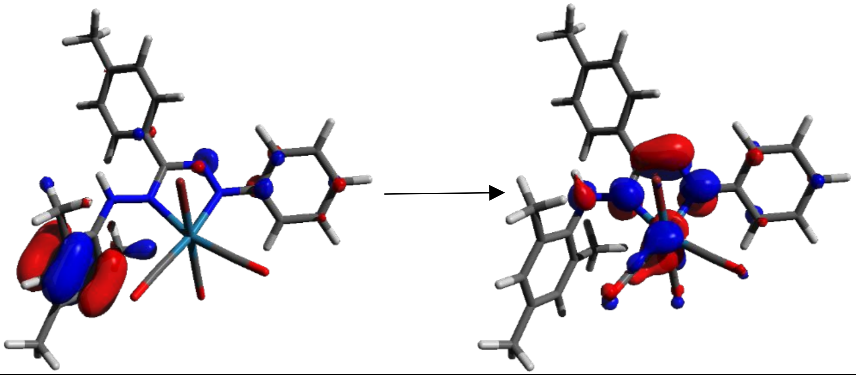	0.98627

9	H-13 → L<br>H-10 → L<br>H-9 → L<br>H-8 →L<br>H-6 → L<br>H-4 → L<br>H-3 → L	329.64/3.7612		0.1694	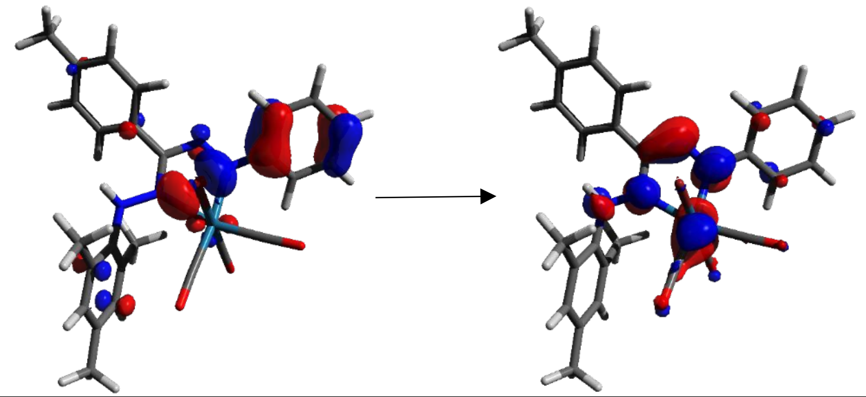	0.97723

a)

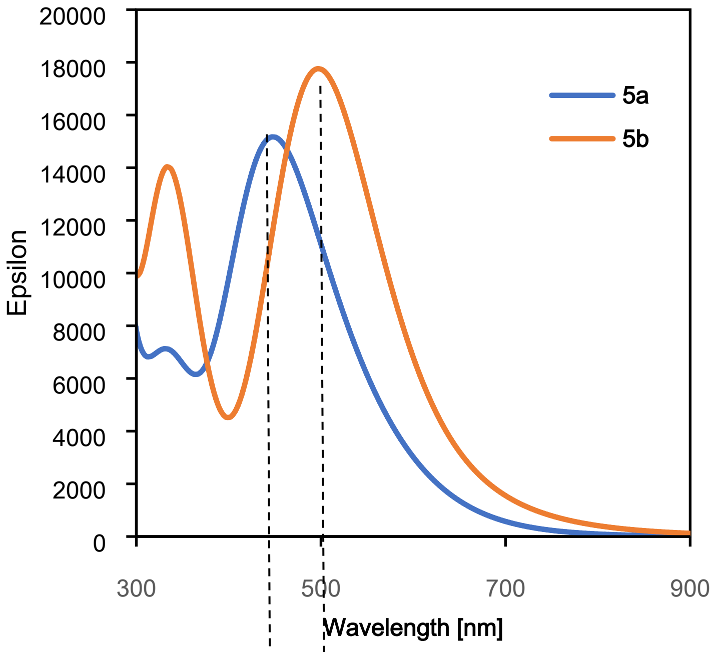

b)

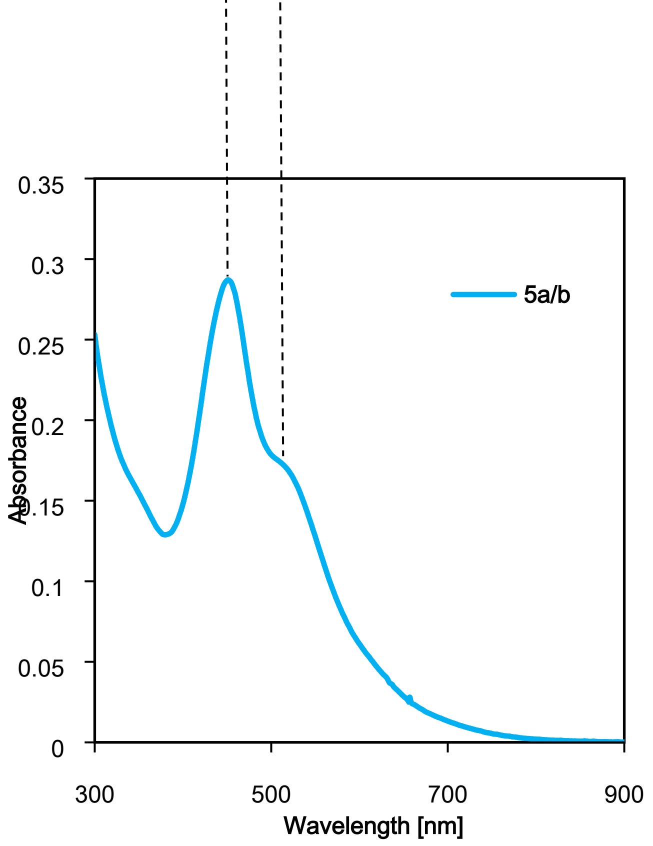

Figure S16. Comparison between the a) theoretical spectra of 5a/b and b) the experimental spectrum.

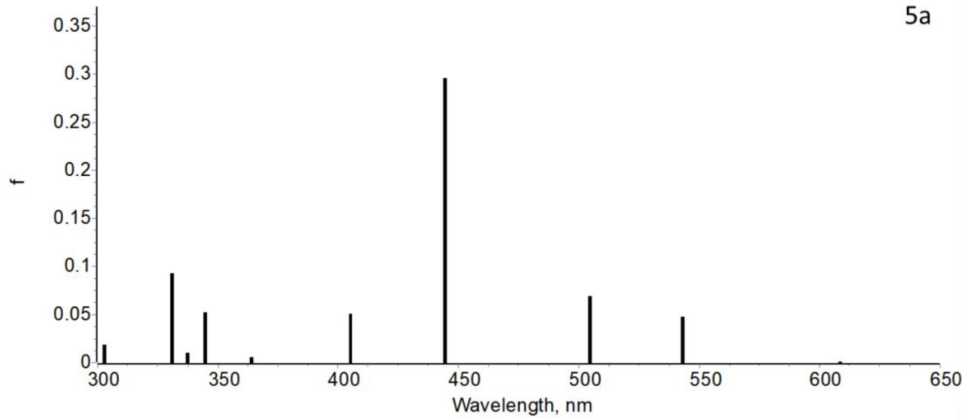

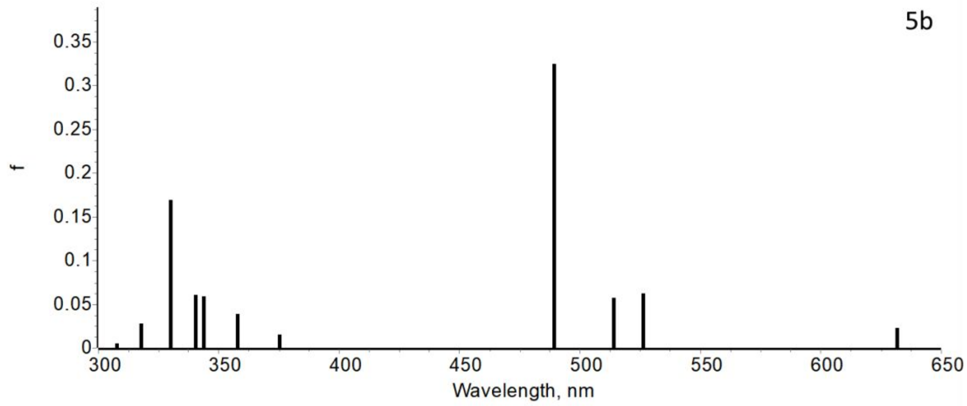

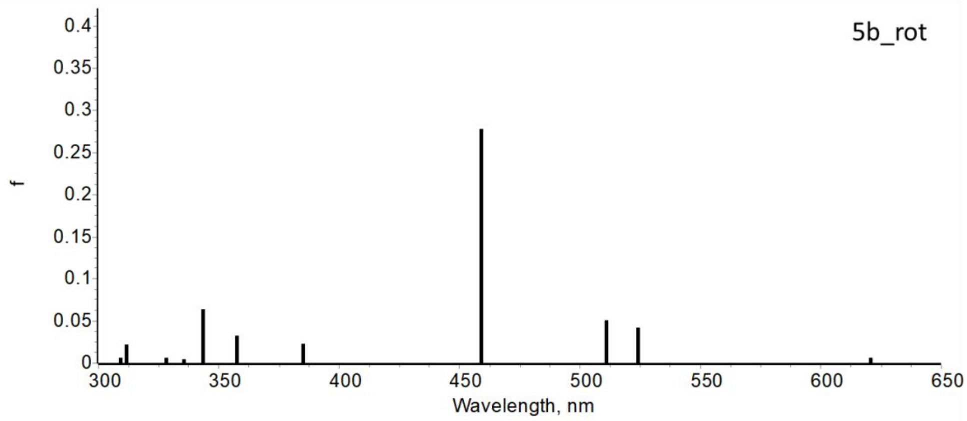

Figure S17. Comparison between the TDDFT-calculated electronic transitions in 5a, 5b and 5b_rot shown between 300 and 650 nm. Structure 5b_rot has the N-Ph group rotated to match the orientation found in 5a. The structure below shows an overlay of 5b (yellow) and 5b_rot (green)

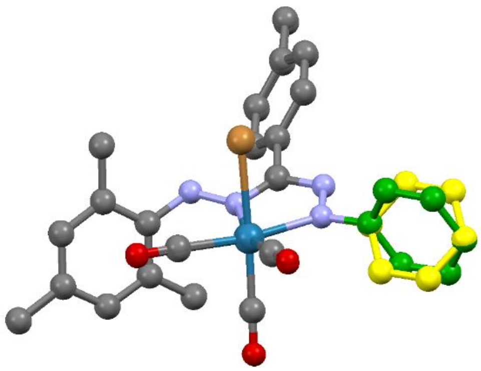

Fragment-based NBO analysis

Using Gaussian16, a CAM-B3LYP/def2-TZVP calculation was performed, followed by an NBO analysis [A.E. Reed, F. Weinhold, Natural bond orbital analysis of near-Hartree-Fock water dimer, J. Chem. Phys. 78, 4066-4073 (1983)]. The NBOs were subsequently divided in NBOs localized on the azo-bound Ph ring, the connecting bond between the Ph ring and the rest of the complex. The Fock matrix elements between NBOs localized on different fragments were set to 0.0, and was diagonalized to find the orbitals of the complex with no interaction between the Ph-ring and the rest of the molecule. The original Fock matrix was then transformed to the basis of the non-interacting MOs, and was diagonalized to obtain the MOs of the complex in the basis of the fragment MOs.

**5b**					**5b_rot**			
MO	C=N	Re	phenyl		MO	C=N	Re	phenyl
H-3	0.3	98.9	0.8		H-3	0.3	91.2	8.5
H-2	0	92.9	7.1		H-2	0	98.5	1.5
H-1	0	99.4	0.6		H-1	0	99.8	0.2
H	0	95.3	4.7		H	0	99.7	0.3
L	0	89.5	10.5		L	0	98	1.9
L+1	0	87.9	12.1		L+1	0	95.5	4.5

Table S7. Orbital mixing (percentage contributions) between the azo-bound Ph ring and the rest of the complex in the geometries 5b and 5b_rot (only the orbitals involved in excited state 4 are shown).

- **Studies on luminescence of complex 4**

Luminescence spectroscopy was performed with a *Fluorolog*®*-3* spectrometer from *HORIBA Jobin Yvon.* A four-window cuvette from *Hellma*® *Analytics* (d = 1 cm) was used. A *TBX Picosecond Photon Detection Module* from *HORIBA* Jobin Yvon served as detector. In time-dependent luminescence spectroscopy a *Nano-LED pulsed diode light source* (λ_{max} = 370 nm) from *HORIBA Scientific* combined with a *Single Photon counting controller FluoroHub* from *HORIBA Jobin Yvon* as a pulse control unit were used. Luminescence decay data were corrected for scattered light by a separate measurement of a non-emissive silica suspension at equal excitation and detection wavelength (λ = 370 nm). Lifetimes were obtained from mono exponential fits of the luminescence decay using fit options implemented in *originPro*® from *OriginLab*®. Excitation in steady state luminescence spectroscopy was performed using a *Xenon Short Arc Lamp* from *Ushio Inc*. light source.

a)

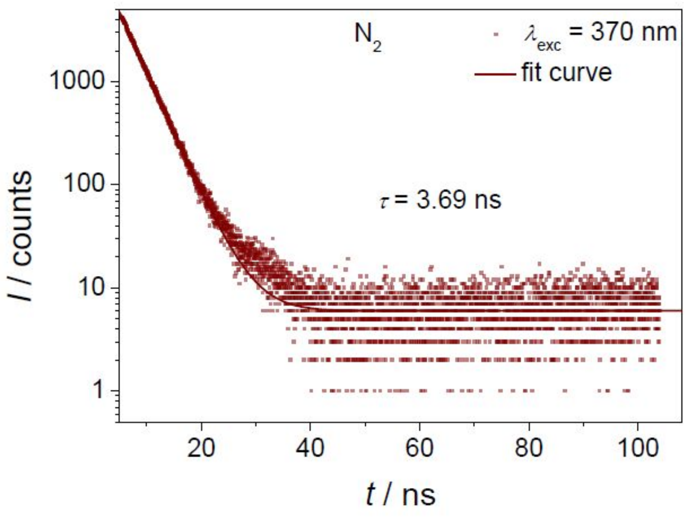

b)

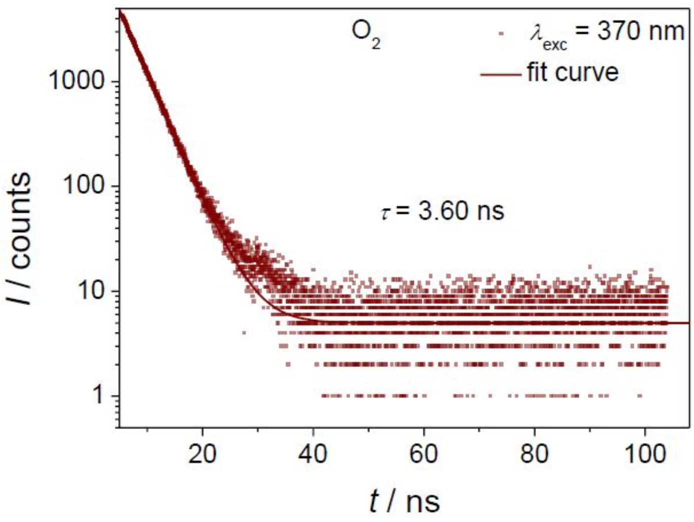

Figure S18. Lifetime decay for 4 in a) under N_2 and b) under O_2 atmosphere.

References

(1) Sae-Heng, P.; Tantirungrotechai, J.; Tantirungrotechai, Y. Scale Factors for Carbonyl Vibrational Frequencies: A Study of Partial Hessian Approximation. *Chiang Mai J. Sci.* **2018**, *45* (7), 2797–2808.
